# Lipopolysaccharide distinctively alters human microglia transcriptomes to resemble microglia from Alzheimer's disease mouse models

**DOI:** 10.1242/dmm.049349

**Published:** 2022-10-18

**Authors:** Jimena Monzón-Sandoval, Elena Burlacu, Devika Agarwal, Adam E. Handel, Liting Wei, John Davis, Sally A. Cowley, M. Zameel Cader, Caleb Webber

**Affiliations:** ^1^ UK Dementia Research Institute, Cardiff University, Cardiff CF24 4HQ, UK; ^2^ John Radcliffe Hospital, Nuffield Department of Clinical Neurosciences, University of Oxford, Oxford OX3 9DS, UK; ^3^ Nuffield Department of Medicine Research Building, Alzheimer's Research UK Oxford Drug Discovery Institute, University of Oxford, Oxford OX3 7FZ, UK; ^4^ Weatherall Institute of Molecular Medicine, University of Oxford, Oxford OX3 9DS, UK; ^5^ James Martin Stem Cell Facility, Sir William Dunn School of Pathology, University of Oxford, Oxford OX1 3RE, UK; ^6^ Translational Molecular Neuroscience Group, New Biochemistry Building, Nuffield Department of Clinical Neurosciences, University of Oxford, Oxford OX1 3QU, UK

**Keywords:** iPSC-microglia, Alzheimer's disease, ATPγS, IFN-γ, LPS, PGE_2_

## Abstract

Alzheimer's disease (AD) is the most common form of dementia, and risk-influencing genetics implicates microglia and neuroimmunity in the pathogenesis of AD. Induced pluripotent stem cell (iPSC)-derived microglia (iPSC-microglia) are increasingly used as a model of AD, but the relevance of historical immune stimuli to model AD is unclear. We performed a detailed cross-comparison over time on the effects of combinatory stimulation of iPSC-microglia, and in particular their relevance to AD. We used single-cell RNA sequencing to measure the transcriptional response of iPSC-microglia after 24 h and 48 h of stimulation with prostaglandin E2 (PGE_2_) or lipopolysaccharide (LPS)+interferon gamma (IFN-γ), either alone or in combination with ATPγS. We observed a shared core transcriptional response of iPSC-microglia to ATPγS and to LPS+IFN-γ, suggestive of a convergent mechanism of action. Across all conditions, we observed a significant overlap, although directional inconsistency to genes that change their expression levels in human microglia from AD patients. Using a data-led approach, we identify a common axis of transcriptomic change across AD genetic mouse models of microglia and show that only LPS provokes a transcriptional response along this axis in mouse microglia and LPS+IFN-γ in human iPSC-microglia.

This article has an associated First Person interview with the first author of the paper.

## Teaser

**Summary:** Using single-cell RNA sequencing to measure the induced pluripotent stem cell-derived microglia response, we find convergence between ATPγS and lipopolysaccharide (LPS)+IFN-γ; however, comparison to genetic mouse models indicates LPS as a better model for Alzheimer's disease.

## INTRODUCTION

Microglia have well-established roles in inflammation, phagocytosis and brain homeostasis, appear to promote neuronal survival during early development (Ueno et al., 2013), participate in synaptic pruning (Paolicelli et al., 2011) and regulate neuronal excitability (Badimon et al., 2020). Microglia constantly survey and react to changes in their environment. The normal functioning of microglia is key to brain homeostasis, while their functional disruption, prolonged activation or ageing may contribute to pathological conditions (Luo et al., 2010). Age-related morphological changes in human microglia include the loss of fine branches and cytoplasmic fragmentation (Streit et al., 2004), and transcriptomic changes such as the upregulation of the amyloid beta (Aβ) formation pathway and the downregulation of the TGFβ pathway (Olah et al., 2018). Genes associated with a higher risk of developing Alzheimer's disease (AD) are significantly associated with microglia-specific expression patterns (Agarwal et al., 2020), and gene expression analyses also highlight key roles for microglia in AD (Zhang et al., 2013; Mukherjee et al., 2019) and other neurodegenerative diseases.

As neuroimmune cells, microglia respond to a large variety of stimuli (Cho et al., 2019), including lipopolysaccharide (LPS), interferon gamma (IFN-γ), prostaglandin E2 (PGE_2_) and ATP studied here. The bacterial endotoxin, LPS, is a potent pro-inflammatory stimulus for microglia and activators of innate immunity. IFN-γ is a soluble cytokine predominantly released from T cells and natural killer cells (Mosser and Edwards, 2008). It is known to regulate leukocyte migration (Reyes-Vazquez et al., 2012) and has elevated expression in models of injury and pathology of the nervous system (Roselli et al., 2018). IFN-γ primes microglia, resulting in changes in morphology and the release of pro-inflammatory cytokines, to thereby heighten microglial responses to other stimuli including LPS. For example, the combination of LPS+IFN-γ potentiates the response of murine macrophages by increasing nitric oxide production (Lowenstein et al., 1993; Held et al., 1999). PGE_2_ is an endogenous lipid immune modulator that elicits diverse functions through binding to different types of EP receptors (EP1, increasing Ca^2+^; EP2 and EP4, increasing cAMP; and EP3, reducing cAMP) (Kawahara et al., 2015). Activation of the PGE_2_/EP2 pathway can promote inflammation in diverse models of neurodegeneration (Liang et al., 2008; Shie et al., 2005; Jin et al., 2007), and targeting EP2 with agonists aims to reduce inflammation, restore healthy microglia function (Amaradhi et al., 2020) and even improve age-related cognitive decline (Minhas et al., 2021). However, the activation of the PGE_2_/EP4 pathway has shown anti-inflammatory effects in Aβ models of AD (Woodling et al., 2014), leading to a dual PGE_2_ function that can be context dependent (Andreasson, 2010; Caggiano and Kraig, 1999). PGE_2_ is also known to exert its effects in other cell types, for example by promoting astrocyte proliferation (Zhang et al., 2009). ATP is released as a transmitter by both neurons (Pankratov et al., 2006; Bodin and Burnstock, 2001) and astrocytes (Guthrie et al., 1999; Anderson et al., 2004; Lalo et al., 2014), but also acts to signal damage when released from injured cells (Rodrigues et al., 2015) and in response to hypoxia (Melani et al., 2005). Extracellular ATP induces microglial chemotaxis both *in vitro* and *in vivo* (Davalos et al., 2005; Ohsawa et al., 2007). The microglial response to external ATP is proposed to be mediated through P2 purinergic receptors (Walz et al., 1993), while the ATP-dependent release of ATP in microglia and astrocytes is suggested as a mechanism to mediate the long-range migration of microglia toward sites of injury (Dou et al., 2012).

Although the effects of inflammatory stimuli on their own have been investigated, changes in response over time, the consequences of combined inflammatory activation in human models and, importantly, their utility for the study of AD are less well explored. To model inflammatory effects, we used human induced pluripotent stem cell (iPSC)-derived microglia (iPSC-microglia), following a highly efficient protocol that broadly recapitulates microglia ontogeny from primitive embryonic macrophages from the yolk sac (Haenseler et al., 2017a,b; Buchrieser et al., 2017). We took advantage of cellular indexing of transcriptomes and epitopes by sequencing (CITE-seq) (Stoeckius et al., 2017) to simultaneously measure, at a single-cell resolution, the transcriptional response of iPSC-microglia to diverse stimuli [LPS+IFN-γ, PGE_2_ and adenosine 5′-O-(3-thio)triphosphate (ATPγS)] after different exposure times. We confirmed the relevance of challenged iPSC-microglia as models for AD, by finding both a higher than expected overlap with genes that change their expression in microglia from AD patients and an unusually high number of protein interactions with the products of genes within AD genome-wide association study (GWAS) loci. We also performed a meta-analysis of microglia from mouse models of AD, identifying a disease axis along which microglia from wild-type (WT) and transgenic AD mouse models are consistently separated. We observed segregation between homeostatic and activated response microglia (ARM) along the disease axis, as well as a minor shift from microglia of post-mortem AD patients. This framework singles out LPS as the only insult we tested that shifts the transcriptional profile of microglia towards a disease state in both mouse and human iPSC-microglia.

## RESULTS

We set out to study the response of iPSC-derived microglia to a series of individual and combined stimuli. More importantly, we investigated whether the iPSC-microglia *in vitro* response is relevant for AD by focusing on both human and mouse models of the disease.

### Individual homogeneous populations of iPSC-microglia show consistent responses to stimuli across biological replicates

We exposed iPSC-microglia to either ATPγS (1 mM), LPS+IFN-γ (10 ng/ml) or PGE_2_ (500 nM), and measured the transcriptional response after 24 h and 48 h. Additionally, iPSC-microglia were exposed to either PGE_2_ or LPS+IFN-γ, with ATPγS added after 24 h and the combined response measured after a further 24 h (Fig. S1A). Prior Ca^2+^-imaging experiments in iPSC-microglia demonstrated that pre-treatment with either PGE_2_ or LPS+IFN-γ for 24 h led to an increased response to ATPγS (Fig. S2). We therefore sought to investigate how treatment with these inflammatory stimuli may alter microglia molecular networks. Across a total of eight conditions, and across four biological replicates, the transcriptional response was measured at the single-cell level using CITE-seq for multiplexing (Stoeckius et al., 2017). All comparisons were made to 0 h controls (untreated).

**Fig. 1.**
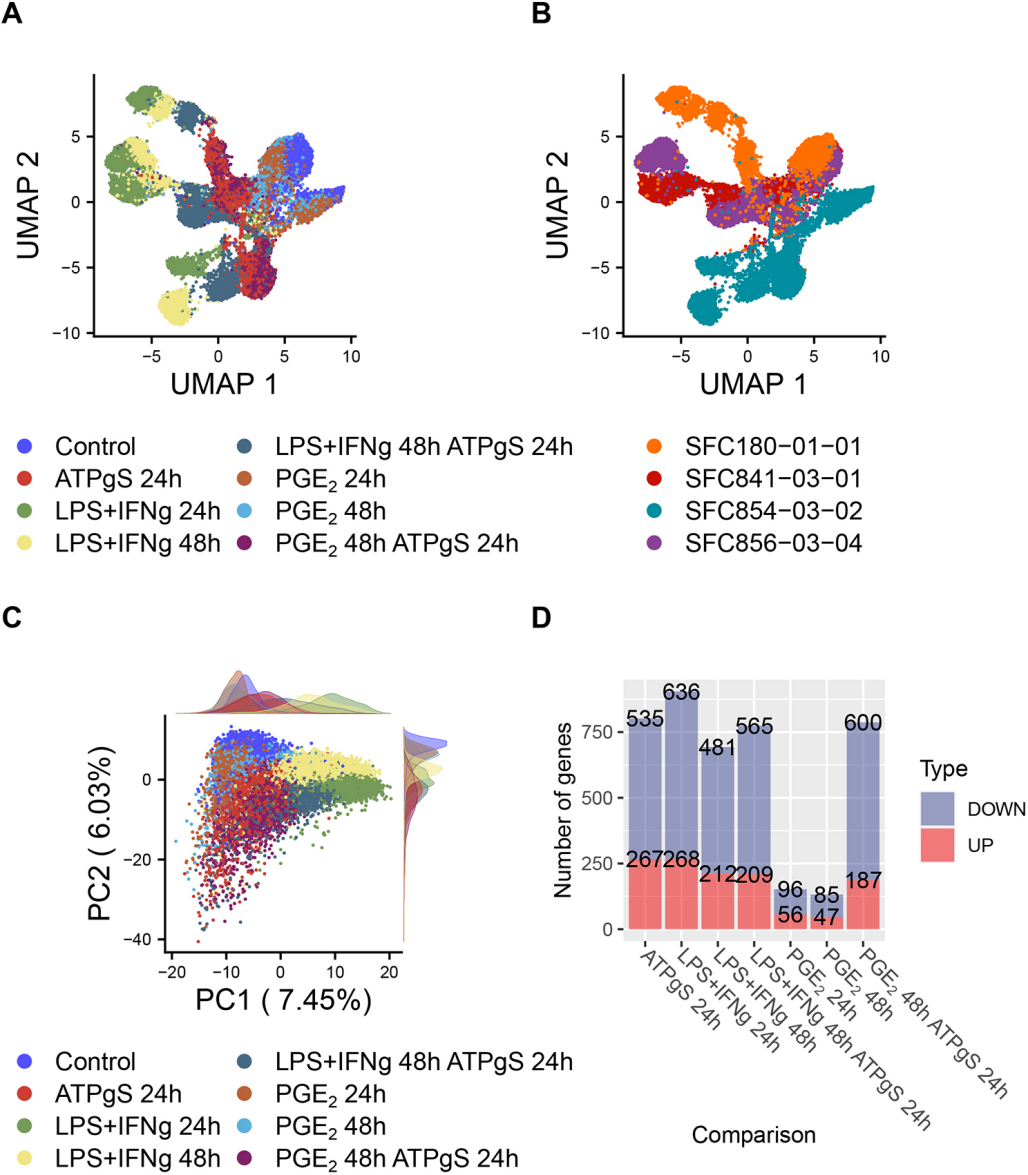
**Induced pluripotent stem cell (iPSC)-derived microglia show a similar response to treatments across biological replicates, with the largest response to lipopolysaccharide (LPS)+interferon gamma (IFN-γ).** (A) Uniform Manifold Approximation and Projection (UMAP) based on the first ten principal components of the top 1000 most variable genes across iPSC-microglia shows segregation of groups exposed to either LPS+IFN-γ or adenosine 5′-O-(3-thio)triphosphate (ATPγS), while those treated only with PGE_2_ tend to cluster near controls. (B) UMAP shows a similar segregation pattern across biological replicates (colours indicate the donors from which iPSC-microglia were derived). (C) Principal component analysis based on the top 1000 most variable genes in iPSC-microglial cells (*n*=19,460). In the first two principal components (PCs), cells are coloured by experimental group; density plots on the side help to distinguish groups treated with LPS+IFN-γ along the first component and groups treated with ATPγS along the second component. (D) Number of differentially expressed genes (DEGs) detected between each treatment and control cells (integrated data, combined *P*-value<0.05).

After de-multiplexing, we obtained the transcriptome of 20,231 single cells and performed unbiased clustering analysis to identify cells with similar transcriptional profiles (see Materials and Methods). We detected eight cell clusters (Fig. S1B) that segregated cells by experimental condition and by donor-to-donor differences (Fig. S1C), with the exception of a small cluster of 469 cells (cluster 6) that did not express microglial markers but appeared to be a fibroblast-like cell population (Fig. S3B, Fig. S4A). We further detected a small population of proliferating microglia (cluster 7, *n*=302 cells) (Fig. S3C, Fig. S4B). We excluded both fibroblast-like cells and proliferating microglia from further analysis. In the remaining microglia-like populations, we observed a consistent transcriptional response across biological replicates upon exposure to the same stimuli (Fig. 1A,B). iPSC-microglia treated with LPS+IFN-γ could be further segregated by time of exposure (24 h and 48 h), while iPSC-microglia treated with ATPγS (either alone or in combination with other stimuli) clustered separately, indicating global similarity within treatments that converge across biological replicates. However, the expression profiles of cells treated with PGE_2_ were more similar to those of untreated control cells, suggesting a milder response.

### Functional convergence of differentially expressed genes (DEGs) after 24 h stimulation with ATPγS and LPS+IFN-γ

**Fig. 2.**
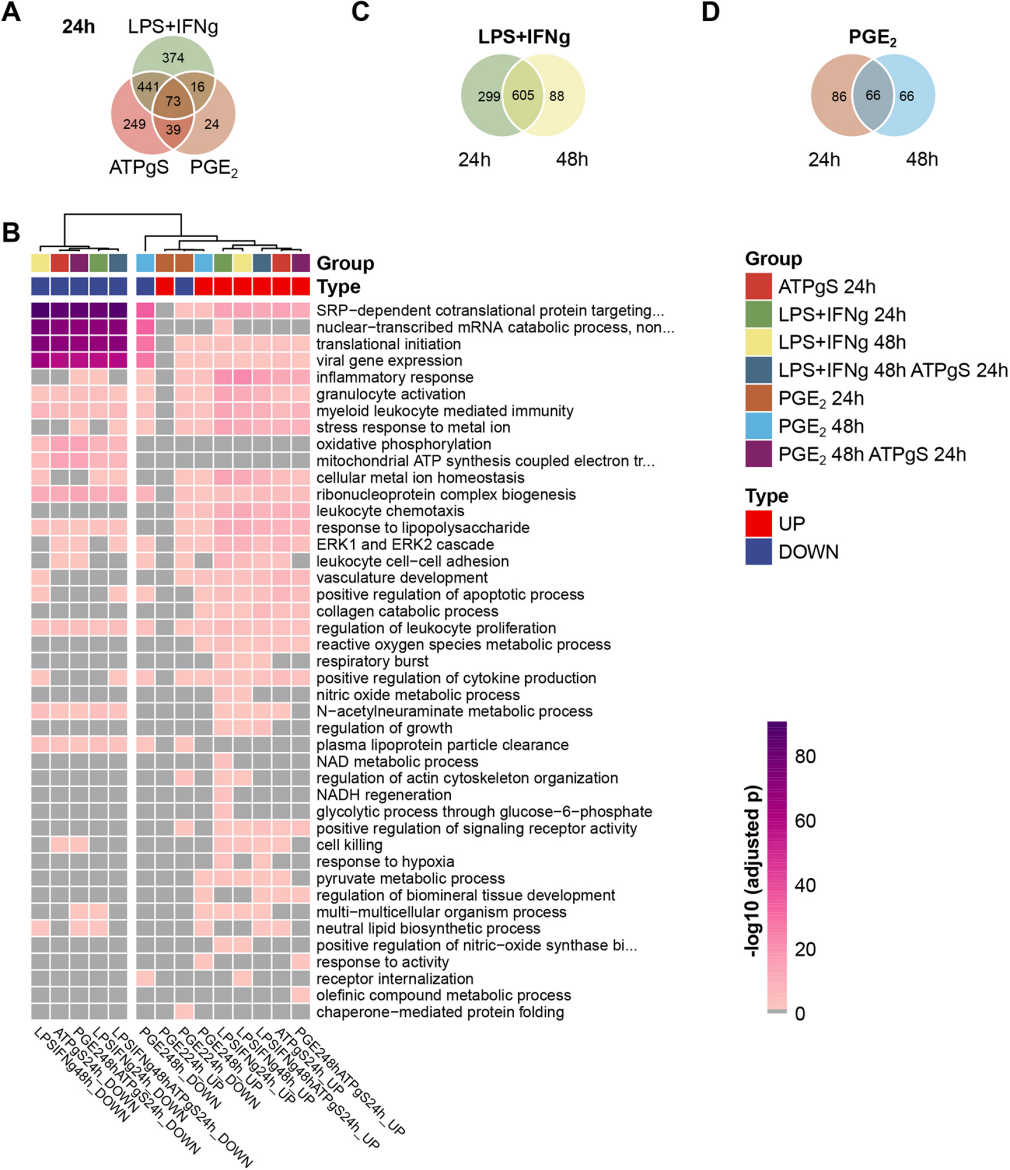
**Consistent increased expression of chemotaxis-related genes and decreased expression of genes involved in translation and signal recognition particle (SRP)-dependent co-translational protein targeting to membrane.** (A) Venn diagram shows the overlap between DEGs (combined *P*-value<0.05) at 24 h after exposure to LPS+IFN-γ, PGE_2_ and ATPγS. (B) Enriched biological processes found among DEGs detected after 24 h and 48 h in response to ATPγS, LPS+IFN-γ and PGE_2_. Gene Ontology enrichment analysis was performed separately for upregulated and downregulated genes. Heatmap shows the −log10-transformed adjusted *P*-value for each enriched biological process in shades of pink (if adjusted *P*-value<0.05, otherwise grey). Only non-redundant terms based on their semantic similarity are shown (see Materials and Methods). (C) Venn diagram shows a larger set of genes uniquely differentially expressed in response to LPS+IFN-γ at 24 h compared to 48 h. (D) Similarly, a larger set of unique DEGs in response to PGE_2_ was found at 24 h.

Principal component analysis (PCA) showed separation of iPSC-microglia treated with LPS+IFN-γ along the first component (7.45% of the variance) and of iPSC-microglia treated with ATPγS along the second component (6.03% of the variance) (Fig. 1C). Given the observed clustering per donor even within control iPSC-microglia (Fig. S5), we integrated our gene expression data across donors (Fig. S6) and performed differential expression analysis grouping by donor (see Materials and Methods). The largest number of DEGs was found after 24 h exposure to LPS+IFN-γ (*n*=904, combined *P*-value<0.05), closely followed by the 24 h stimulation with ATPγS (*n*=802, combined *P*-value<0.05). Fewer gene expression changes were found in response to PGE_2_ after 24 h exposure (*n*=152, combined *P*-value<0.05, Fig. 1D). Despite the wide range of DEGs detected in response to the different stimuli (LPS+IFN-γ, PGE_2_ and ATPγS) and the distinct principal components (PCs), we found a set of 73 overlapping DEGs at 24 h across all treatments (Fig. 2A, hypergeometric test pairwise comparisons; LPS+IFN-γ and ATPγS, *n*=514, *P*≈0, log(*P*)=−1007.81; LPS+IFN-γ and PGE_2_, *n*=89, *P*=8.23×10^−62^; ATPγS and PGE_2_, *n*=112, *P*=4.51258×10^−101^). In particular, the strongest correlation between the gene expression fold changes (FCs) at 24 h was observed between the exposure to LPS+IFN-γ and to ATPγS (*r*=0.625, *P*<2.2×10^−16^, Fig. S7), suggesting a convergent mechanism between these two different stimuli.

Using Gene Ontology (GO) annotations and controlling for the microglia-like gene background, we found strikingly similar sets of enriched biological processes across DEGs, which broadly segregated between upregulated and downregulated genes. However, enriched GO terms from downregulated genes with PGE_2_ tended to cluster with GO terms from upregulated genes in response to the other stimuli. In particular, we found high similarity between the ATPγS treatment and the LPS+IFN-γ treatment at 24 h compared to controls (Fig. 2B). Among downregulated genes, in response to both ATPγS and LPS+IFN-γ at 24 h, we found enrichment of genes associated with reduced gene expression, including genes involved in translational initiation, nuclear-transcribed mRNA catabolic process nonsense-mediated decay, signal recognition particle (SRP)-dependent co-translational targeting to membrane, oxidative phosphorylation, mitochondrial ATP synthesis and plasma lipoprotein particle clearance. Genes involved in the immune response were enriched among both upregulated and downregulated genes in response to both ATPγS and LPS+IFN-γ, but only among downregulated genes in response to PGE_2_. Notably, enrichment of genes involved in the cellular response to LPS, as well as the IFN-γ-mediated signalling pathway, was found among upregulated genes with ATPγS, again pointing towards a common mechanism in the iPSC-microglia response to ATPγS and to LPS+IFN-γ. In contrast, among the upregulated DEGs in response to PGE_2_ at 24 h, there was no enrichment of genes already implicated in the response to LPS alone.

### Distinct temporal gene expression patterns in response to LPS+IFN-γ versus PGE_2_

The DEGs in response to LPS+IFN-γ at both 24 h and 48 h following exposure were more similar to each other than to the DEGs in response to PGE_2_ across the same time points. Specifically, when comparing the sets of DEGs in response to LPS+IFN-γ at both 24 h and 48 h, we observed a higher overlap (*n*=605, Jaccard index=0.609, hypergeometric test, *P*≈0, log(*P*)=−1707.006, Fig. 2C) than in response to PGE_2_ (*n*=66, Jaccard index=0.303, hypergeometric test, *P*=1.484×10^−95^, Fig. 2D). Although similar biological processes were enriched at both 24 h and 48 h in response to LPS+IFN-γ, direct comparison between the two time points revealed that a fraction of DEGs at 24 h are returning to baseline at 48 h (Fig. S8C,E), and thus DEGs show opposite directions from 0 h to 24 h and from 24 h to 48 h. In contrast, when we compared the response to PGE_2_ at 24 h and 48 h, we found biological processes uniquely enriched at each time point. For example, in contrast to ATPγS and LPS+IFN-γ, inflammatory response genes were downregulated 24 h after PGE_2_ treatment, but, 48 h after exposure, pathways shared with ATPγS and LPS+IFN-γ were also enriched among genes that are differentially expressed in response to PGE_2_, including upregulated regulation of the IFN-γ production pathway and inflammatory response and downregulation of genes involved in nuclear-transcribed mRNA catabolic processes (Fig. S8E). Our results show that although LPS+IFN-γ provokes a broad, intense and transient response, PGE_2_, by contrast, has a reduced but more complex and in some aspects delayed response, consistent with its dual pro-inflammatory and anti-inflammatory role (Fig. S8D,E).

### Lack of widespread synergistic effects of the combined treatments with ATPγS

Although ATPγS treatment alone provoked a strong cellular response, little additional effect was observed when this treatment was combined with the prolonged exposure of either LPS+IFN-γ or PGE_2_ (Fig. S9). Specifically, only 20 DEGs were uniquely identified in response to the combined treatment of LPS+IFN-γ 48 h and ATPγS at 24 h compared to ATPγS alone (Fig. S9B), while only two unique DEGs were found in response to the combined effect of 48 h PGE_2_ and 24 h ATPγS compared to ATPγS alone (Fig. S9D). Additionally, in the combined treatments with ATPγS, we found almost the same set of biological pathways once we controlled for the effects of the individual treatments (Fig. S9I). We observed similar FCs in response to LPS+IFN-γ at 48 h with and without the addition of ATPγS at 24 h, whereas only strong changes were observed in response to the combined treatment of PGE_2_ at 48 h with the addition of ATPγS (Fig. S9E,F). Gene expression changes in response to the combined treatment of PGE_2_ and ATPγS were quite similar to those observed in response to ATPγS alone (Fig. S9H). Taken together, these findings suggest the lack of widespread synergistic effects of LPS+IFN-γ or PGE_2_ treatments when either treatment is combined with ATPγS.

### Combined protein–protein interaction (PPI) network highlights a core similar response to LPS+IFN-γ and to ATPγS

**Fig. 3.**
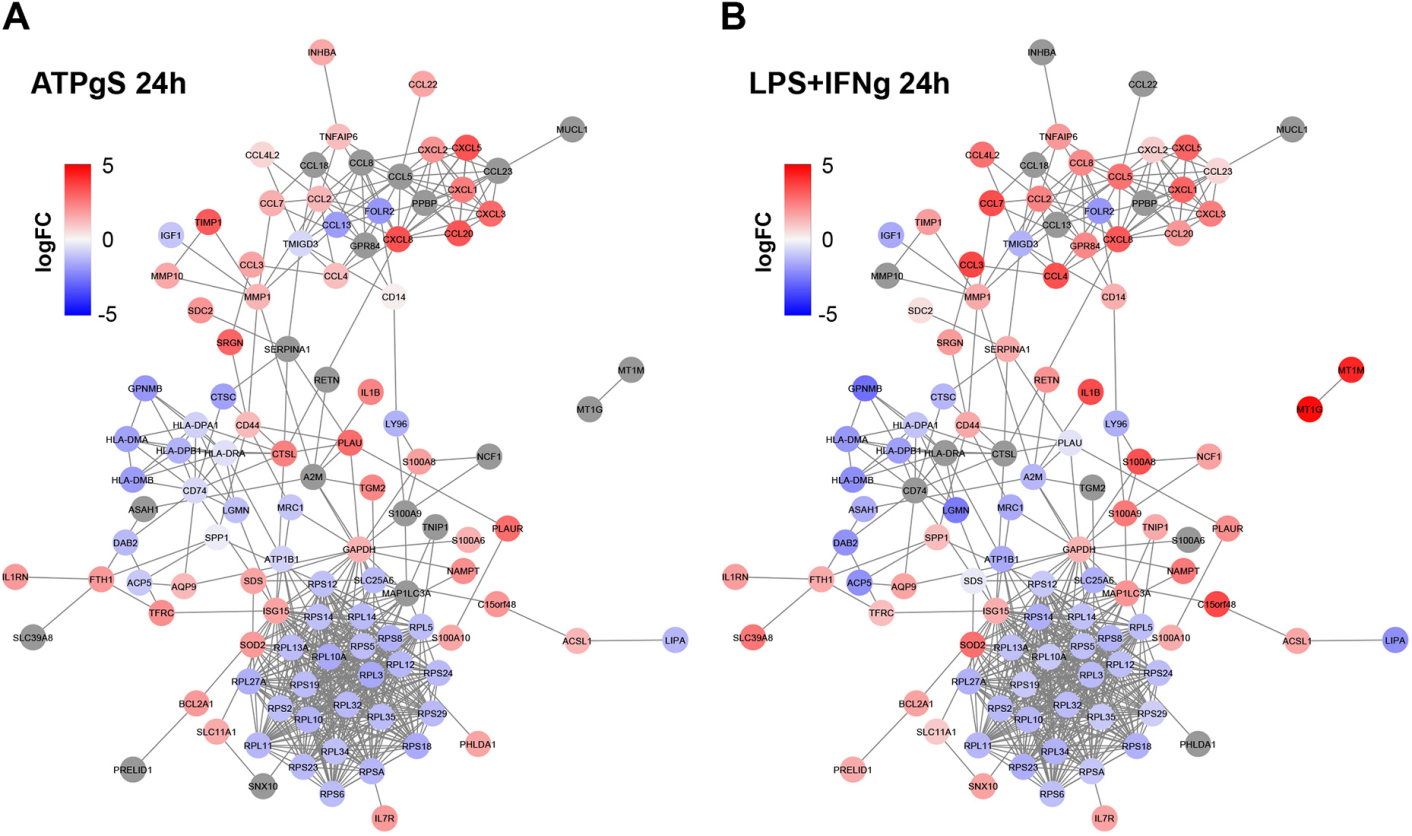
**Functional convergence among ATPγS and LPS+IFN-γ treatments at 24 h through a combined protein–protein interaction (PPI) network.** Nodes indicate genes, and edges indicate known PPIs between their gene products (see Materials and Methods). PPI network among the protein products of the DEGs with the largest fold changes (FCs) in any of the treatments (absolute logFC≥1.5, combined *P*-value<0.05). (A) Genes are coloured by the logFC after 24 h in response to ATPγS. (B) Genes are coloured by the logFC after 24 h in response to LPS+IFN-γ.

Using an integrated PPI network (see Materials and Methods), we found more interactions than expected by chance among the protein products of the DEGs in iPSC-microglia in response to each of the different stimuli once we controlled for degree and gene length (estimated *P*-value<0.0001 based on randomizations, see Materials and Methods, Fig. S10A). These results further support the functional convergence within each set of identified DEGs. By focusing on a subset of the PPI network containing the genes with the most marked changes at 24 h (absolute logFC>=1.5, combined *P*-value<0.05), we observed a high level of similarity in the direction and strength of the gene expression changes upon ATPγS and LPS+IFN-γ, in addition to functional clustering among upregulated and downregulated genes (Fig. 3; Fig. S7).

### DEGs in iPSC-microglia across all treatments significantly overlap with genes that change in microglia of AD patients

**Fig. 4.**
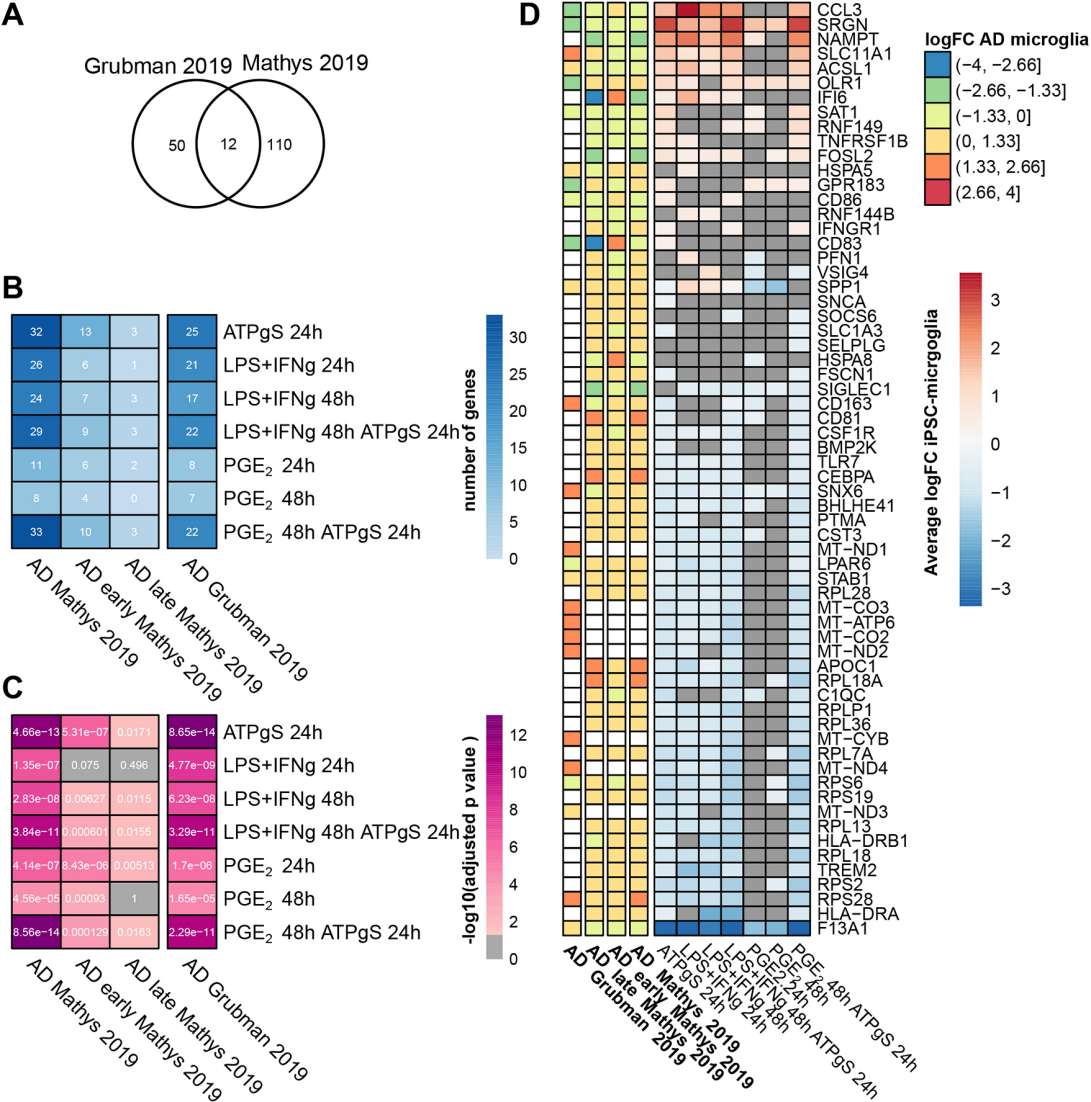
**Overlapping DEGs in stimulated iPSC-microglia and DEGs in microglia from Alzheimer's disease (AD) patients.** (A) Venn diagram shows the overlap between DEGs in microglia from AD patients identified by Mathys et al. (2019) in the prefrontal cortex and by Grubman et al. (2019) in the entorhinal cortex (hypergeometric test, *n*=12, *P*=4.525×10^−12^). (B) Heatmap shows the number of overlapping iPSC-microglia DEGs and the DEGs in microglia from AD patients. It includes the subsets of DEGs that change in expression early in the pathology of AD (contrasting individuals that showed amyloid burden but few neurofibrillary tangles and modest cognitive impairment) and the subset of genes that change late in the pathology of AD (higher amyloid burden, presence of neurofibrillary tangles and cognitive impairment compared to the early pathology group). (C) We tested whether the overlap between DEGs was higher than expected by chance; the heatmap indicates the adjusted *P*-value of the corresponding hypergeometric tests (adjusted *P*-value<0.05 shown in pink shades, otherwise shown in grey). (D) Heatmap shows the direction and magnitude of the change (logFC) of the DEGs in iPSC-microglia and the DEGs in microglia from AD patients; grey squares indicate no significant change in expression (adjusted *P*-value>0.05).

In AD, a large fraction of risk genes are highly expressed in microglia compared to other cell types, and efforts to characterize cell-type-specific transcriptional changes from post-mortem tissue of patients with AD have been recently reported (Mathys et al., 2019; Grubman et al., 2019). Grubman et al. (2019) characterized cell-specific gene expression changes from the entorhinal cortex of six patients with AD and six controls, while Mathys et al. (2019) focused on cell-specific changes in the prefrontal cortex of 24 individuals with AD and 24 controls. Both reported microglial-specific changes in AD patients compared to controls (62 DEGs in the entorhinal cortex and 122 DEGs in prefrontal cortex, Fig. 4A). Although there is heterogeneity between AD datasets, the overlap of 12 genes between datasets is higher than expected by chance (hypergeometric test, *P*=4.525×10^−12^). We compared the transcriptional changes in our challenged iPSC-microglia and the microglia-specific changes observed in both post-mortem AD studies (see Materials and Methods). We observed a small, but higher than expected, overlap between the genes differentially expressed in iPSC-microglia following all challenges and the DEGs in microglia from AD patients from both studies, including genes that change in the early state of the pathology (Fig. 4B,C). However, differences were observed in the direction of the effect. In the iPSC-derived stimulated microglia, most of the gene expression changes occurred in the same direction [such as the upregulation of serglycin (*SRGN*)]. Only a few genes showed divergent expression patterns, such as secreted phosphoprotein 1 (*SPP1*) upregulation with LPS+IFN-γ and downregulation with PGE_2_ treatments. Another discordant example was the chemokine (C-C motif) ligand 3 (*CCL3*), upregulated only in response to ATPγS and LPS+IFN-γ but not in response to PGE_2_ (Fig. 4D). By contrast, we observed more changes in gene expression in opposite directions when comparing the challenged iPSC-microglia to the post-mortem microglia of AD patients. For example, mitochondrial and ribosomal genes were downregulated in iPSC-microglia and upregulated in the post-mortem AD microglia. Thus, we perturb a small but significant subset of genes altered in post-mortem AD microglia when challenging iPSC-microglia with different stimuli. Although few differences in directionality are observed between iPSC-microglia challenged with these different stimuli, larger differences in directionality exist between these challenged iPSC-microglia and post-mortem AD microglia.

### DEGs in iPSC-microglia are linked through PPIs to genes that change in microglia of AD patients and to AD GWAS risk genes

Using the combined PPI network, we found more PPIs than expected by chance between DEGs in post-mortem AD microglia and DEGs in our stimulated iPSC-microglia, suggesting functional convergence into shared pathways [PPIs controlled for cell-type-specific effects, coding sequence (CDS) length and node degree, see Materials and Methods, Fig. S11A]. We also found more PPIs between genes lying within AD GWAS risk loci and each of every set of DEGs in challenged iPSC-microglia (Fig. S11B). The functional links between the *in vitro* perturbations in iPSC-microglia and the genetic risk of developing AD, as well as the post-diagnosis gene expression changes observed in post-mortem AD, suggest that all these challenged iPSC-microglia could be relevant models for AD study.

### Meta-analysis of mouse microglia allows the identification of a disease axis that segregates WT microglia from transgenic AD model microglia

**Fig. 5.**
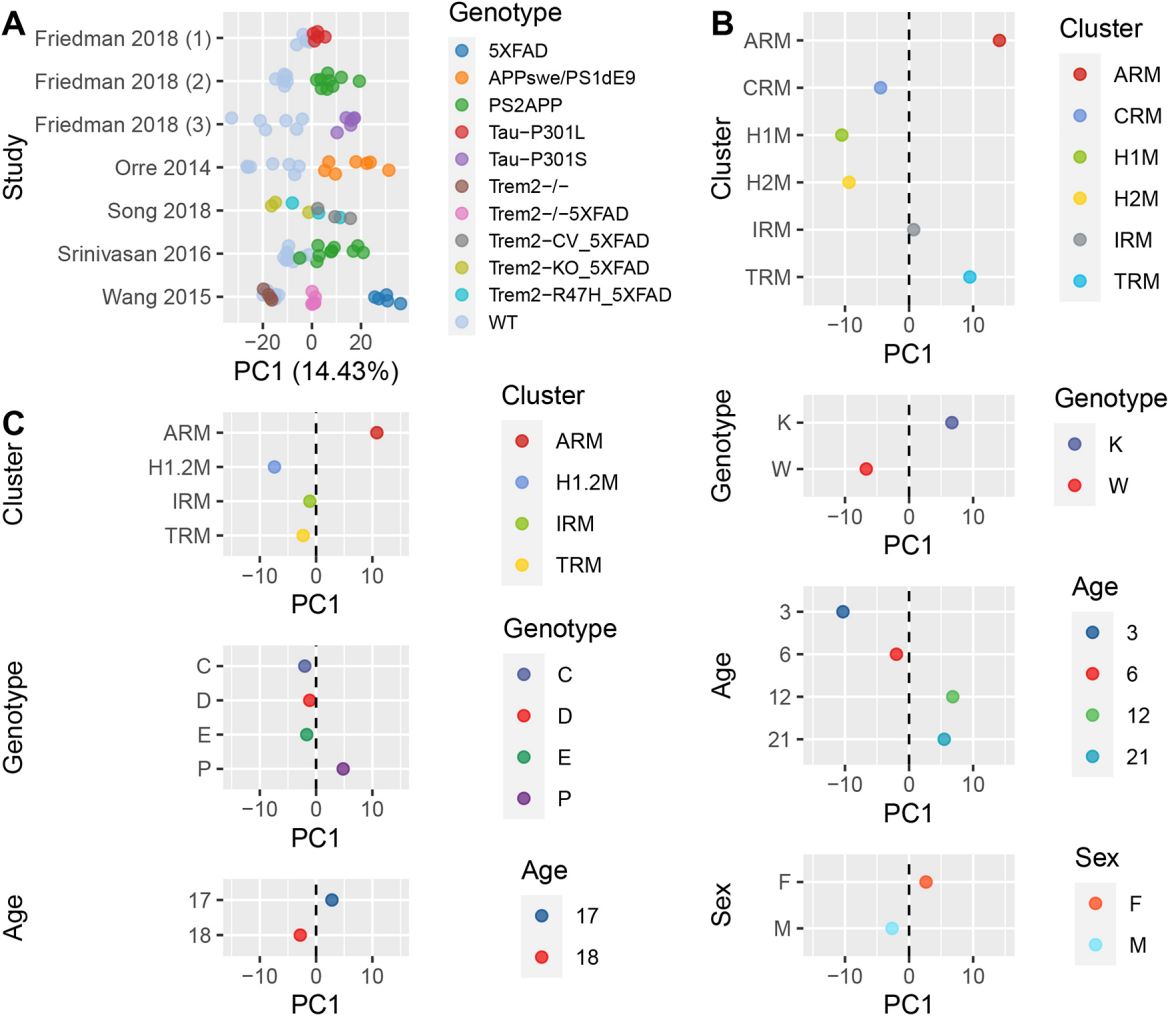
**Disease axis from meta-analysis of microglia from genetic mouse models of AD segregates homeostatic and activated response microglia.** (A) After accounting for batch effects, first PC segregates mouse microglia from WT and from those of transgenic mouse models of AD across datasets. (B) Single-cell gene expression of microglia from the knock-in *App^NL-G-F^* and WT mice was aggregated by either microglia type/cluster (ARM, activated response microglia; CRM, cycling/proliferating microglia; H1M, homeostatic microglia 1; H2M, homeostatic microglia 2; IRM, interferon response microglia; TRM, transit response microglia), genotype (K, *App^NL-G-F^*; W, WT), age (3, 6, 12 and 21 months) or sex (F, female; M, male) and projected into the first PC or disease axis. Each dot represents the projected PC1 for the aggregated transcriptional profile of microglia across 10,187 shared genes. (C) Single-cell gene expression of microglia from male WT and APP/PS1 mice was aggregated by either microglia type/cluster (H1.2M, homeostatic microglia 1/2), genotype C, C57BL/6; D, *App*/*Ps1*-*Apoe*KO; E, C57BL/6-*Apoe*KO; P, *App*/*Ps1* or age (17 and 18 months) and projected into the disease axis from the meta-analysis of mouse AD models.

As a final comparison for our challenged human iPSC-microglia, we compared them to *in vivo* purified microglia across a range of published AD mouse models. Although a small fraction of AD-relevant risk genes lack a 1:1 human: mouse orthologue (Mancuso et al., 2019), genetic mouse models are useful as they allow the study of behaviour and cognitive decline, and recapitulate some physiopathological features of the disease. We performed a gene expression meta-analysis of purified mouse microglia across a series of transgenic models of AD including genetic mutations in amyloid precursor protein (*APP*), presenilin (*PS1*; also known as *PSEN1*), microtubule-associated protein tau (*MAPT*) and triggering receptor expressed on myeloid cells 2 (*TREM2*) (Wang et al., 2015; Song et al., 2018; Orre et al., 2014; Srinivasan et al., 2016; Friedman et al., 2018). After data re-processing and accounting for batch effects (see Materials and Methods), the first PC (accounting for 14.43% of the variance) segregated WT from transgenic models carrying genetic mutations associated with AD (Fig. 5A; Fig. S12A-C). We refer herein to the first PC as the disease model axis. Along this data-driven disease axis, the most severe model (5xFAD) showed the most segregation, while microglia with a TREM2 knockout clustered with WT microglia.

Next, we asked whether the orthologues of genes lying within AD GWAS risk loci were enriched among the genes driving the gene expression differences along the disease axis (see Materials and Methods). From 116 AD GWAS loci genes, we identified 55 with one-to-one orthologue correspondence from human to mice expressed across the microglia gene datasets used in the meta-analysis. However, when we focused on the top 500 genes with the lowest loadings along the disease model axis (corresponding to a reduced expression in AD models), we found more AD GWAS loci genes than expected by chance (hypergeometric test, *P*=0.00197), including *HBEGF*, *CASS4*, *OARD1*, *CNN2*, *IL6R*, *BZW2*, *BIN1*, *FRMD4A* and *ADAM10* (Fig. S12D). We confirmed the overlap in different-sized windows, from the top 50 to 1000 genes in 50 gene increments. A significant overlap with AD GWAS loci genes held true when testing the top 200-300, 400-750 and 900-1000 genes with lowest loadings (adjusted *P*-value<0.05). We also found more PPIs to AD GWAS loci genes than expected by chance in the top genes with the highest and lowest loadings along the disease model axis (Fig. S12E). Genes with the highest PC1 loadings showed enrichment of genes involved in the innate immune response (including response to bacteria), regulation of cytokine production and Aβ clearance, whereas genes with the lowest loadings along PC1 showed enrichment of genes involved in the positive regulation of defence response, negative regulation of cell proliferation and blood vessel morphogenesis (Fig. S13). In summary, the meta-analysis of mouse microglia revealed a disease model axis of microglia gene expression variation that aligns with the disease severity observed in the genetic mouse models of AD, where genes driving the differences along this axis are enriched in AD GWAS loci genes, have more PPIs to AD GWAS loci genes than expected by chance and are enriched in pathways relevant to the disease models of AD.

### Disease axis from genetic mouse models of AD segregates homeostatic from ARM

We next asked whether the disease model axis could also segregate the recently reported ARM subtypes/states that localize with Aβ accumulation in the transgenic mouse APP knock-in model (*App^NL-G-F^*) (Sala Frigerio et al., 2019). We re-normalized and aggregated gene expression data of the *App^NL-G-F^* mouse model by either microglia subtype, genotype, sex, age or tissue, and projected the transcriptional profiles into the disease axis created from the meta-analysis of mouse models of AD (see Materials and Methods, Fig. 5B). We observed that the largest segregation along the disease axis occurred when we compared homeostatic microglia, which localized as microglia from WT in other studies, and ARM, which localized similarly to AD model microglia. To a lesser degree, we also observed segregation along the disease axis by genotype, age and sex, in agreement with previous observations in which microglia from female mice progress more rapidly to an ARM state (Sala Frigerio et al., 2019). Differences between homeostatic and ARM microglia along the disease axis were further confirmed when projecting analogous gene expression data from the APP/PS1 mouse model reported in the same study (Fig. 5C). In this second dataset, we also observed that APOE knockout moved microglia along the disease axis towards a transcriptional profile more similar to that of the WT, consistent with previous observations where its deletion prevents the main inflammatory response to Aβ plaques (Sala Frigerio et al., 2019).

### LPS treatment shifts the transcriptional profile of microglia towards a disease state

**Fig. 6.**
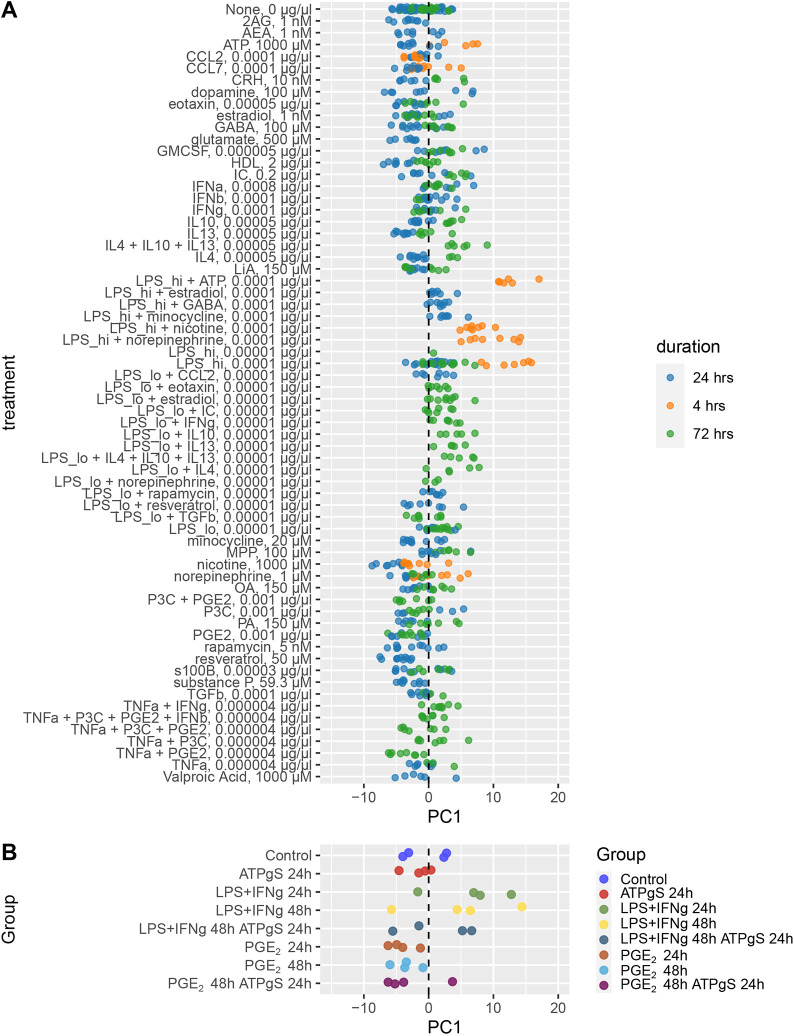
**LPS shifts mouse primary microglia and human iPSC-microglia towards a more similar profile to that of mouse models of AD.** (A) Gene expression data of *in vitro* mouse microglia stimulated with a large array of different stimuli were projected into the disease axis (or first PC based on the meta-analysis of gene expression of microglia from genetic mouse models of AD). Each dot represents the projected PC1 based on the transcriptional profile of 10,844 shared genes. Largest shifts along PC1 occur in microglia treated with high doses of LPS at 4 h. (B) Gene expression data from our human iPSC-microglia were aggregated by experimental group and donor and projected into the disease axis (PC1) from the meta-analysis of microglia from genetic models of AD. Each dot represents the projected PC1 based on the transcriptional profile of 8156 common genes. iPSC-microglia treated with LPS+IFN-γ showed the largest shift along the PC1 projection. We used randomization analysis (see Materials and Methods) to test whether the average rank of treatments that included LPS along PC1 was higher than expected by chance. In mouse primary microglia and in human iPSC-microglia, samples treated with LPS ranked higher along PC1 (estimated *P*-values: *P*_Mouse_<1×10^−5^, *P*_Human_=0.00208).

Following the data-led establishment of a framework that segregates at the transcriptional level WT microglia from mouse genetic AD model microglia, and that captures differences between homeostatic and ARM subtypes/states, we then asked which different inflammatory stimuli, if any, drive microglia along this disease model axis towards a transcriptional state similar to that observed in the disease models of AD. To this end, we re-analysed the transcriptional profiles recently reported (Cho et al., 2019) that systematically assess the microglia response to an array of stimuli across 96 different conditions. Once we accounted for batch effects, we projected each treated microglia transcriptional profile onto the disease model axis (see Materials and Methods). We observed that, after 4 h treatment with high doses of LPS, microglia transcriptional profiles showed the largest shift along the disease axis (Fig. 6A). Similarly, we created a pseudo-bulk from our human iPSC-microglia, averaging expression per donor and per treatment, based only on those genes with one-to-one orthologue correspondence between species, accounting for batch effects, and projected the transcriptional profiles into the disease axis (see Materials and Methods). Again, only iPSC-microglia treated with LPS+IFN-γ shifted along the disease axis (Fig. 6B). We further performed a randomization analysis in which we ranked all samples along PC1 and tested whether the transcriptional profiles of microglia stimulated with LPS ranked higher along PC1 than expected by chance. In mouse primary microglia and in human iPSCs, we observed a higher ranking along PC1 in microglia treated with LPS (estimated *P*-values: *P*_Mouse_<1×10^−5^, *P*_Human_=0.00208). Finally, we encountered a large overlapping set of functional pathways shared among the upregulated genes in response to LPS+IFN-y and those with the highest loadings along the disease axis (Fig. S13B). Taken together, these results indicate that, despite the core similarities observed in response to ATPγS and to LPS+IFN-γ, it is the response to LPS by both mouse microglia and human iPSC-microglia that best promotes a transcriptional shift towards a state more similar to that of the ARM from the mouse AD models.

### Minor shift along disease axis of human post-mortem microglia from AD patients

Finally, we projected the gene expression profiles of human post-mortem microglia from individuals with AD and healthy controls (Mathys et al., 2019; Grubman et al., 2019) onto the disease model axis created (Fig. S14). We observed a small but consistent shift along the disease axis, where transcriptional profiles of microglia from individuals with AD segregate along the disease axis closer to the transgenic models of AD, and those from controls towards the profiles of microglia from WT mouse.

## DISCUSSION

In this study, we compared the transcriptomic response of iPSC-microglia to a range and combination of different stimuli at different exposure times and then asked whether any of these challenges provoked a cellular response that that could be useful when modelling AD. Our single-cell approach allowed us to remove contaminating fibroblast-like cells and proliferating microglia and focus on the large fraction of iPSC-microglia (Fig. S3). We showed a consistent response to the different stimuli across four biological replicates (Fig. S6), where the main sources of variation correspond to the exposure type (Fig. 1C), with LPS+IFN-γ and ATPγS provoking the largest number of transiently DEGs (Fig. 1D) with the strongest functional convergence in terms of shared enriched biological pathways, compared to a milder but more complex response to PGE_2_ (Figs 2, 3; Fig. S8). Few additional effects were observed when combining treatments, which supports both functional convergence and dominance of individual effects (Fig. 1D; Fig. S9).

In comparison to microglia, for nuclei obtained from human post-mortem AD, although there is a significant overlap in the DEGs (Fig. 4B,C), the direction of change is largely not concordant (Fig. 4D). This lack of agreement on direction could reflect the temporal nature of immune stimulation (Fig. 2; Fig. S8) and that the post-mortem microglia are likely to be far more neuropathologically heterogeneous than the comparatively controlled and homogeneous iPSC-microglia challenges. In terms of convergent biological processes, across all iPSC-microglia treatments, we found significantly more protein–protein interactors than expected by chance to either DEGs in microglia from AD patients or to genes lying in AD GWAS loci (while controlling for the microglial background), indicating that by stimulating iPSC-microglia we are perturbing gene networks functionally associated with AD.

To further pursue the question of relevance of iPSC-microglia models to AD, we employed an unbiased approach to reveal a shared axis of gene expression variation that distinguished purified WT microglia from AD model microglia across a wide range of AD mouse models (Fig. 5). The discovered axis reflects large and small shifts in gene expression across a great many genes, rather than a smaller number of independently statistically significant changes in a subset of genes. Human post-mortem microglia showed a consistent but small change along the disease model axis, separating AD cases from controls. The lack of a stronger segregation of human post-mortem microglia along the microglia disease axis from AD mouse models might reflect distinct biology or differences in comparative timing and heterogeneity in the transcriptional profiles of AD post-mortem microglia. Placing the gene expression profiles from all human *in vitro* iPSC-microglia challenges and all *in vitro* mouse microglia challenges considered in this study onto this disease model axis singled out the expression changes invoked by LPS in mouse and LPS+IFN-γ in human iPSC-microglia as the challenge that distinctively produces a gene expression reaction similar to that shared among AD genetic mouse models (Fig. 6). Nevertheless, given the lack of stimulation of LPS alone in iPSC-microglia, we were unable to confirm the result using LPS alone in human.

Although, overall, we observed a great similarity between the response to ATPγS and to LPS+IFN-γ, suggesting shared mechanisms of action, we speculate that key differences could be operating upstream of these shared mechanisms that may shift the transcriptional profile towards a state resembling that of the mouse disease models of AD. As observed in mice, LPS alone is able to shift the transcriptional profile of microglia towards a more AD disease model state whereas IFN-γ alone does not induce this shift (at least at the observed times/doses) (Fig. 6A). Although it would be of interest to test the effect of other stimuli, for example Aβ fibrils, our current results propose that, from the stimuli we tested, LPS provokes the most AD-relevant microglia stimulus given its similarity to the genetic mouse models. LPS also has advantages in terms of assay reproducibility, availability and scalability. Although LPS is not known to, nor likely to, cause AD, the Toll-like receptor 4 that mediates the LPS response is thought to have a role in AD (Park and Lee, 2013; Calvo-Rodriguez et al., 2020).

Our data-led approach to identifying an AD disease model transcriptional axis for microglia can be revisited with new model data and further investigated for disease insight. Although there is a strong agreement between the response to LPS and the genetic mouse models of AD, for most of the overlapping DEGs, the directionality of change is not consistent with human post-mortem microglia. Noticeably, an exception, *SPP1* was among the top genes driving the shift along the disease axis from the genetic mouse models of AD, was exclusively upregulated in the iPSC-microglia treated with LPS+IFN-γ at both 24 and 28 h, has increased expression in two different studies of post-mortem human AD microglia (Grubman et al., 2019; Mathys et al., 2019) and is characteristic of the ARM subtype (Sala Frigerio et al., 2019). A microglia population expressing *Spp1* has been described in the axon tracts of the pre-myelinated brain during early post-natal development in mouse (Hammond et al., 2019) and has also been associated with a specific microglia population from a model of toxic demyelination and in human microglia of multiple sclerosis patients (Masuda et al., 2019). The role of *SPP1* both during normal conditions and development and in disease warrants further study.

## MATERIALS AND METHODS

### Cell culture, differentiation and processing

Two male [SFC841-03-01 (Dafinca et al., 2016), SFC854-03-02 (Haenseler et al., 2017a,b)] and two female [SFC180-01-01 (Haenseler et al., 2017a,b), SFC856-03-04 (Haenseler et al., 2017a,b)] iPSC lines were used for the study. They were originally re-programmed from healthy donors recruited through StemBANCC/Oxford Parkinson's Disease Centre [participants were recruited to this study having given signed informed consent, which included derivation of human iPSC lines from skin biopsies; Ethics Committee: National Health Service, Health Research Authority, NRES Committee South Central, Berkshire, UK (REC 10/H0505/71)], and are all listed in hPSCreg and available from the European Bank for Induced Pluripotent Stem Cells (EBiSC). They were differentiated to primitive macrophage precursors and subsequently skewed to microglia-like cells in monoculture according to Haenseler et al. (2017a,b). Primitive macrophage precursors were plated in IBIDI dishes (IBIDI µ-dish 35 mm, low, cat. no. 80136) at a starting density of 500.000 cells per IBIDI dish (∼125,000 cells/cm^2^). Cells were treated with 10 ng/ml LPS and 10 ng/ml IFN-γ or with 500 nM PGE_2_ for 24 h or 48 h in a final volume of 500 µl medium per IBIDI dish; 1 mM ATPγS was added for the second 24 h where relevant (Fig. S1). Note that our experimental design compares all 24 h or 48 h treatments to 0 h controls where relevant, and thus is unable to distinguish *in vitro* changes due only to culturing cells without treatment for 24 h or 48 h.

Cells were lifted by incubating them with 200 µl accutase (Thermo Fisher Scientific) for 3 min at 37°C. Cells were then collected in 2×500 μl PBS and pelleted by spinning at 600 ***g*** for 5 min at 4°C. Next, cells were resuspended in 100 µl staining buffer (2% bovine serum albumin, 0.02% PBS-Tween 20) and incubated with 7 μl Fc blocking reagent (BioLegend) for 10 min. Then 1 µg cell hashing antibodies was added to each of the samples. Each cell line had eight IBIDI dishes corresponding to the eight different treatment conditions and eight hashing antibodies. After 30 min incubation at 4°C, cells were washed two times: first wash by spinning them at 600 ***g*** for 5 min and resuspending them in 500 ml staining buffer spinning, second wash by spinning the cells at 600 ***g*** for 5 min and resuspending them in 200 µl staining buffer. Finally, cells were resuspended in 150 µl PBS, filtered through a 40 µm cell strainer and counted. Note that cultures were staggered and RNA was extracted at the same time to avoid batch effects. All the treatments from a cell line were pooled together and were loaded on a 10X Chromium. For SFC841-03-01, SFC856-03-04 and SFC180-01-01 cell lines, 10,000 cells per pool were loaded in one 10X Chromium lane; for SFC854-03-02 cell line, 5000 cells per pool were loaded on two 10X Chromium lanes.

### Ca^2+^ imaging

For ratiometric Ca^2+^ imaging, microglia from the male line (SFC841-03-01) were incubated in aCSF (130 mM NaCl, 25 mM NaHCO_3_, 2.5 mM KCl, 1.25 mM NaH_2_PO_4_, 2 mM CaCl_2_, 1 mM MgCl_2_ and 10 mM glucose, pH 7.4, 290-310 Osm) containing 5 µM Fura-2 AM and 80 µM pluronic acid (Thermo Fisher Scientific) for 1 h at 37°C after their 24 h pre-treatment incubation. After incubation with Fura-2 AM, the cells were washed with aCSF to remove extracellular dye and left to sit for 30 min at room temperature before imaging. During imaging recording, the first few minutes were recorded with only aCSF. Vehicle or ATPγS (50 µM) was washed on and off the cells in a time-dependent manner. The fluorescence of Fura-2 was excited alternatively at wavelengths of 340 nm and 380 nm by means of a high-speed wavelength-switching device on a Zeiss microscope. Zeiss image analysis software allowed selection of several regions of interest within the field of view. Ratiometric 340/380 calculation was performed with a background subtraction. The 340/380 ratios were then analysed by measuring the average value in a user-defined time window using custom scripts in MATLAB. The data were smoothed using robust local regression MATLAB function at 20%.

### Data processing

We used Cell Ranger pipeline (v2.1.0) to process the sequencing data, including alignment with STAR and single cell 3′ gene counting. CITE-seq-Count python tool was used to de-multiplex samples by hashtag antibody. Then, we used the HTODemux function from Seurat to identify doublets and keep singlets (*n*=20,231). We kept only protein-coding genes detected in at least 100 cells. Thus, we obtained gene expression data for 12,335 genes across 20,231 cells. In the filtered dataset, we observed a median of 2309 genes, 10,293 unique molecular identifiers (UMIs) and 3.25% of mitochondrial reads per cell. Gene expression was normalized against the total number of counts detected per cell. Gene expression data were scaled to a factor of 1×10^4^ before the transformation to logarithmic scale.

### Dimensionality reduction and clustering

We performed PCA on the scaled gene expression of the top 1000 most variable features (across 20,231 cells). For visualization, we used Uniform Manifold Approximation and Projection (UMAP) based on the first 30 PCs. To identify communities of similar cells, we used the shared nearest neighbour (SNN) modularity optimization-based clustering algorithm (FindClusters function in Seurat R package). To identify unbiased clusters, we included the first 20 PCs and a granularity resolution of 0.1. Most cell clusters showed expression of the microglial marker *C1QB* (Fig. S3A), except cluster 6, consisting of 469 cells, which instead showed increased expression levels of *COL1A1* (Fig. S3B) and other common fibroblast markers (Muhl et al., 2020). For a more comprehensive characterization, we considered the expression levels of a core set of 249 human microglial markers identified by Patir et al. (2019) from a meta-analysis of transcriptomic data (Patir et al., 2019). Many human microglial makers were expressed across all cell clusters, except in cluster 6, the fibroblast cell population (Fig. S3C, Fig. S4A). We also detected a small population (cluster 7) of proliferating iPSC-microglia characterized by the expression of *KIAA0101* (also known as *PCLAF*), *UBE2C*, *TOP2A* and *CDK1* (Fig. S4B). We excluded from further analysis both cluster 6 (fibroblast-like) and cluster 7 (proliferating cells) and performed PCA again. Initially PCA was performed only on untreated control iPSC-microglia (*n*=1751), where we used the first 30 PCs for UMAP and clustering (Fig. S5). Then, PCs were re-calculated for iPSC-microglia across all experimental groups (*n*=19,460), and, again, the first 30 PCs were used for UMAP.

### Data integration

We performed an integration step across our biological replicates (donors). Gene expression data were divided into smaller datasets per donor and normalized, and the top 1000 most variable features were identified. A total of 1620 features were repeatedly variable and were used to find anchors. Canonical correlation analyses were performed across each pair of datasets. Integrated data were scaled for PCA. For visualization, we used UMAP based on the first 30 PCs.

### Differential expression

Differential expression analysis was performed in the integrated dataset, using the FindConservedMarkers function in Seurat R package. Each experimental condition was compared to the untreated control cell per donor independently using a Wilcoxon rank sum test. Therefore, each gene was tested four times, one per donor. The metap R package was used to combine *P*-values using the minimump function that implements the Tippett's method, for the meta-analysis of *P*-values. Genes with a combined *P*-value<0.05 were considered differentially expressed.

### GO enrichment

For GO enrichment analyses, we used clusterProfiler R package. GO annotations were accessed through Bioconductor (org.Hs.eg.db). We used as background population the set of genes expressed in our dataset (*n*=12,335). We used false discovery rate (FDR) to account for multiple testing and considered enriched only those terms with an adjusted *P*-value<0.05. To reduce redundancy among enriched GO terms, we used rrvgo R package using *Rel* similarity with a threshold of 0.85. Similarly, GO mouse annotations were accessed through Bioconductor (org.Mm.eg.db). All genes detected in the meta-analysis were considered our background gene population.

### Combined PPI network

We constructed a PPI network based on the data available across a range of resources: BioGRID (Stark et al., 2006) (accessed 30 March 2020), HitPredict (López et al., 2015) (accessed 30 March 2020), IntAct (Orchard et al., 2014) (accessed 30 March 2020), STRING (Szklarczyk et al., 2019) (accessed 30 March 2020, only links with experimental evidence score>0), CORUM (Giurgiu et al., 2019) (accessed 30 March 2020), Reactome (Fabregat et al., 2018) (accessed 30 March 2020), BioPlex HCT116.v.1.0 (accessed 30 March 2020), BioPlex 3.0 (Huttlin et al., 2021) (accessed 30 March 2020), MINT (Licata et al., 2012) (accessed 30 March 2020), InBioMap (Li et al., 2017) (accessed 30 March 2020). All PPIs were either kept or mapped to Ensembl gene IDs. When we tested whether the number of PPIs was higher among a set of genes than expected by chance, we performed 10,000 randomizations. In each randomization, we selected an equally sized sample of genes matched for degree and CDS length and counted the number of PPIs among them. An estimated *P*-value was derived from the number of randomizations where we detected more PPIs than observed among the protein products of each set of DEGs.

### Test for gene overlap

We used a hypergeometric test for the overlap between each pair of sets of DEGs. We adjusted for multiple testing using the Benjamini–Hochberg method. We used as background a population of 12,335 genes to estimate the expected proportions. When we compared *Homo sapiens* and *Mus musculus*, only genes with one-to-one orthologue correspondence were taken into account.

### Microglia response to diverse stimuli in mice

We re-processed the gene expression data from mouse microglia exposed to 96 different conditions *in vitro* available at Gene Expression Omnibus (GEO) [GSE109329 (Cho et al., 2019)]. We quantified transcript abundances using Kallisto version kallisto_linux-v0.46.0 (Bray et al., 2016). The reference index was built based on coding (cdna) and non-coding RNA (ncrna) sequences with annotations from Ensembl release 98 available through the ftp website (http://ftp.ensembl.org/pub/release-98/fasta/mus_musculus/cdna/Mus_musculus.GRCm38.cdna.all.fa.gz; http://ftp.ensembl.org/pub/release-98/fasta/mus_musculus/ncrna/Mus_musculus.GRCm38.ncrna.fa.gz). We filtered out sequences in scaffold chromosomes. We filtered genes with no expression across all samples. For comparison between species, only genes with one-to-one orthologues from *Homo sapiens* to *Mus musculus* were considered.

### DEGs in human AD patients

We used data from two independent studies that have reported microglia-specific gene expression changes in AD patients compared to controls (Grubman et al., 2019; Mathys et al., 2019). Genes reported by Mathys et al. (2019) (Supplementary Table 2 in their publication, FDR-adjusted *P*-value<0.05, two-sided Wilcoxon rank sum test), and those reported by Grubman et al. (2019) on the accompanying website to their publication (http://adsn.ddnetbio.com/; AD versus control based on subclusters, *n*=62 genes, FDR<0.05, *n*=62 genes, empirical Bayes quasi-likelihood *F*-test), were evaluated.

### Meta-analysis of microglia from genetic mouse models of AD

Gene expression datasets from mouse microglia were obtained from GEO through a search of genetic models of AD (search in GEO for ‘microglia mouse AD’ in 2018). Microarray datasets included the following: fluorescence-activated cell sorting (FACS)-purified microglia from 8.5-month-old WT, *Trem2*^−/−^, 5XFAD, and *Trem2*^−/−^ 5XFAD [GSE65067 (Wang et al., 2015)]; CD45^+^ and CD11B^+^ microglia from 8.5-month-old mice expressing the common variant, R47H or no human TREM2 on a background of murine TREM2 deficiency and the 5XFAD mouse model of AD [GSE108595 (Song et al., 2018)]; and cortical microglia from 15- to 18-month-old APPswe/PS1dE9 mice compared to WT littermates [GSE74615 (Orre et al., 2014)]. RNA-sequencing datasets included the following: FACS-sorted microglia from 7- or 13-month-old PS2APP or non-transgenic mice [GSE75431 (Srinivasan et al., 2016)]; microglia (*Cx3cr1*::GFP^+^ sorted) from the cortex of 14- to 15-month-old PS2APP or WT mice [GSE89482 (Friedman et al., 2018)]; sorted CD11B^+^ myeloid cells from 11- to 12-month-old tau-P301L and non-transgenic littermates [GSE93179 (Friedman et al., 2018)]; and sorted CD11B^+^ myeloid cells from 6-month-old tau-P301S transgenic mice or non-transgenic littermates [GSE93180 (Friedman et al., 2018)]. For RNA-sequencing datasets, fastq files were downloaded from GEO, and transcript quantification was performed with Salmon (version 0.9.1) for protein-coding genes with Ensembl (release v91). Quality control metrics are provided in Table S1. Transcript counts for all studies were imported and summarized to gene levels counts with tximport R library, and genes with less than 20 counts across all samples were filtered out. The filtered count matrix was normalized using Rlog transformation implemented in DESeq2 R library (Love et al., 2014). For microarray datasets, CEL files were downloaded from GEO. We performed background subtraction, quantile normalization and summarization using the RMA algorithm implemented in oligo R library (Carvalho and Irizarry, 2010). Then, we used surrogate variable analysis to correct for batch effects between the seven studies through the ComBat function available in the sva R library (Chakraborty et al., 2012). Finally, we performed PCA using the prcomp function in R.

### Projection into PC1 of mouse genetic AD models meta-analysis

We projected samples from a few datasets into the same dimensional space (PC1) from the meta-analysis created from the genetic mouse models of AD, with one dataset projected at a time. For each dataset we projected into PC1, we corrected for batch effects using ComBat along with the rest of the datasets from the meta-analysis. Then, we centred the batch-corrected data from the dataset to be projected and multiplied it by the gene loadings of PC1 (contained in the rotation slot from the corresponding prcomp object in R). For the single-cell datasets, we averaged gene expression by either experimental group, microglia subtype, genotype, age or sex before correcting for batch effects.

To test whether LPS-stimulated microglia tended to rank higher along PC1, we first ranked all the projected samples along PC1 (separately for mouse microglia, and for human iPSC-microglia). We obtained the average rank for all the samples that included LPS (mouse microglia) or LPS+IFN-γ (iPSC-microglia) and compared it to the average rank of 100,000 equally sized random samples. We obtained an estimated *P*-value by counting the number of times that the random samples had an average higher rank along PC1.

### Mouse microglia subtypes from single-cell gene expression data

Counts were downloaded directly from GEO (GSE127892, GSE127884), and meta-data were extracted from loom files available at scope.bdslab.org (Sala Frigerio et al., 2019). Counts from each dataset (APP/PS1 and APP^NF-G-L^) were normalized and scaled using the logNormalize method with a scale factor of 10,000 implemented in the NormalizeData and ScaleData functions from Seurat R package (Stuart et al., 2019). Gene expression was averaged either by microglia subtype cluster, genotype, age or sex.

### PPIs to AD GWAS risk genes, and to DEGs in microglia of AD patients

From the GWAS catalogue (Buniello et al., 2019), we obtained all mapped genes to single-nucleotide polymorphisms associated with AD traits (EFO_0000249; accessed 16 November 2020, *P*≤1×10^−8^). From the set of 116 AD GWAS risk genes, we found that 72 had expression in our iPSC-microglia and were included in the combined PPI network described above. Then, we tested whether the number of PPIs between each set of DEGs and AD GWAS risk genes was higher than expected by chance. To this end, we contrasted the number of PPIs among the gene products of each set of DEGS in iPSC-microglia to those of 10,000 equally sized random samples from our background population (genes expressed in iPSC-microglia), while controlling for the CDS length and degree of the random sets in the PPI network. An estimated *P*-value was drawn from the 10,000 randomizations. The same approach was used to test whether the number of PPIs between each set of DEGs (iPSC-microglia) and DEGs in microglia of AD patients was higher than expected by chance.

We also tested whether the genes with the top 500 highest and lowest loading along the disease axis (PC1 of the meta-analysis) had more PPIs than expected by chance to AD GWAS genes. In this case, the background population was reduced to genes detected in the meta-analysis that had a one-to-one orthologue relationship from mouse to human.

## Supplementary Material

10.1242/dmm.049349_sup1 Supplementary information
