# Supplementary material for: Lipopolysaccharide distinctively alters human microglia transcriptomes to resemble microglia from Alzheimer's disease mouse models
Source: Dis Model Mech. 2022 Oct 18;15(10):dmm049349. doi: 10.1242/dmm.049349 (PMC9612871; doi:10.1242/dmm.049349)
Supplement: Supplementary information [file dmm-15-049349-s1.pdf]

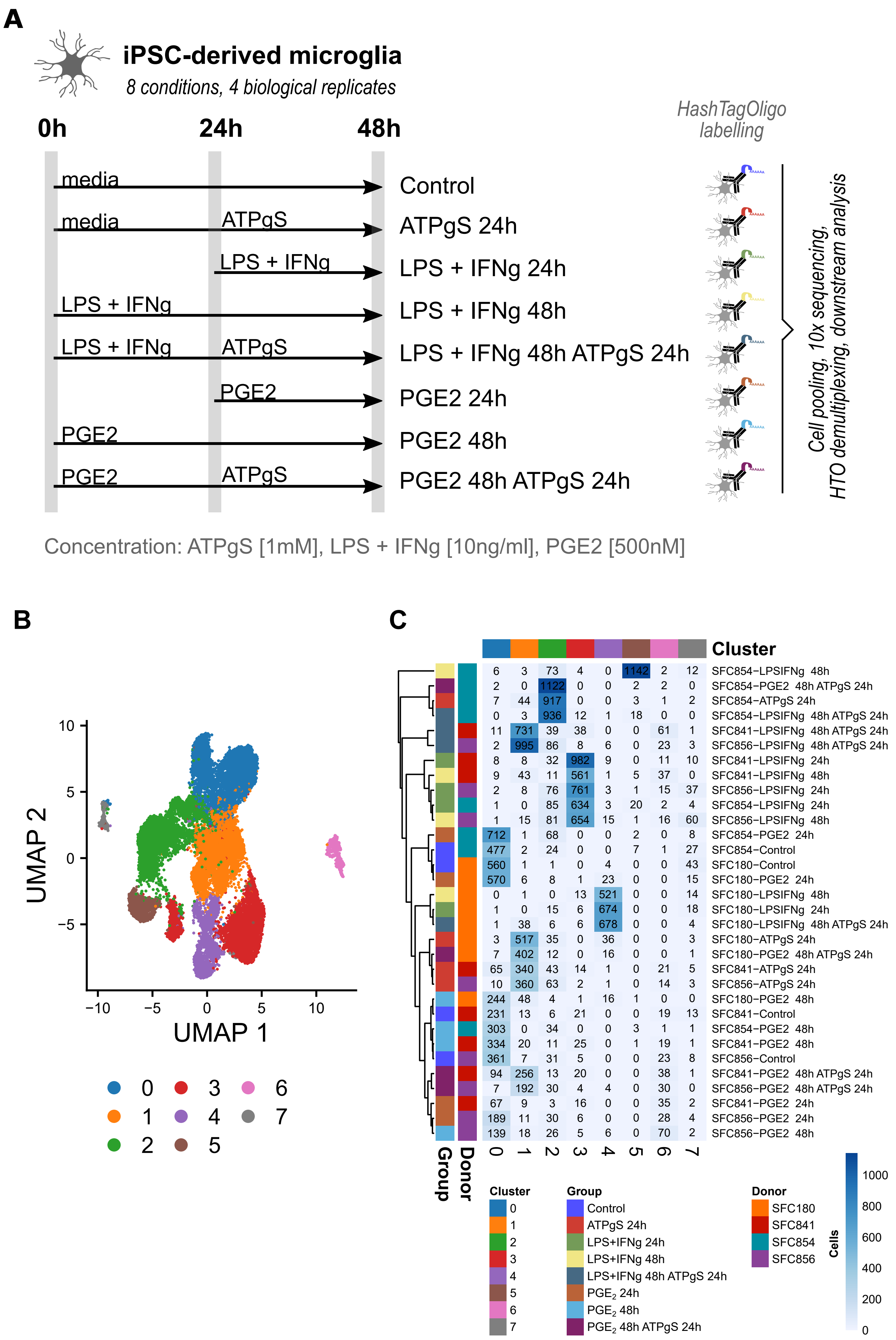

Disease Models & Mechanisms • Supplementary information

Fig. S1. Experimental design to measure transcriptional response of iPSC-microglia and unbiased cell clustering. A iPSC- microglia from 4 different donor were separated into 8 experimental groups : 1) Control iPSC-microglia maintained for 48 hours in normal media, 2) iPSC-microglia exposed to ATPγS [1mM] for 24 hours, 3) iPSC-microglia treated with LPS +IFN-γ [10ng/ml] for 24 hours, 4) iPSC-microglia treated with LPS+IFN-γ [10ng/ml] for 48 hours, 5) iPSC-microglia were exposed to LPS+IFN-γ for 24 hours, and then to ATPγS for an additional 24 hours, 6) iPSC-microglia were exposed to PGE2 for 24 hours. 7) iPSC-microglia exposed to PGE2 for 48 hours and 8) iPSC-microglia primed with PGE2 for 24 hours and then exposed to ATPγS for 24 hours more. We simultaneously measured the transcriptional response at the single cell level with CITE-seq for multiplexing (Stoeckius et al.), in which a particular hashtag oligo (HTO) was used to label iPSC-microglia from each condition. Library preparation and sequencing (10x) were performed, followed by in silico de-multiplexing and data analysis. **B** We detected eight unbiased cell clusters, UMAP plot coloured shows similarity of transcriptional profiles of 20,231 cells coloured by their cluster identity. **C** Heatmap shows the number of cells assigned to each of the eight unbiased clusters annotated to both the donor and the experimental group. Cluster 0 is mainly composed by control and PGE2 treated cells at both 24 and 48h, cluster 1 of cells treated with ATPγS either alone or in combination, cluster 2 has an overrepresentation of cells from donor SFC854, clusters 3 and 4 contain most of cells treated with LPS+IFN-γ, cluster 5 mostly cells from SFC854 treated with LPS+IFN-γ at 48h, cluster 6 and 7 were later characterized as fibroblast-like and proliferating iPSC-microglia respectively (**Fig. S3, S4**).

Disease Models & Mechanisms • Supplementary information

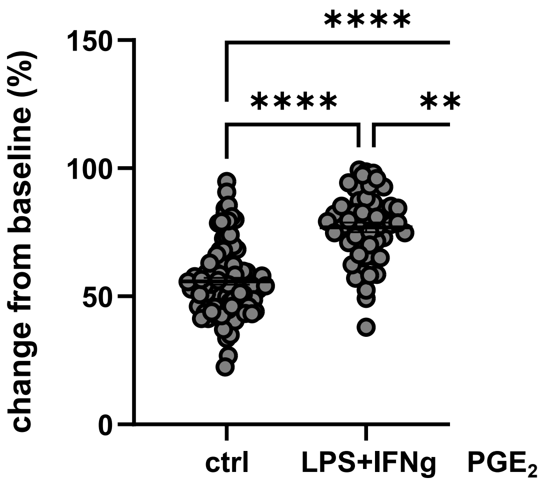

Fig. S2. Percentage of calcium increase, calculated from ratiometric (340/380nm) calcium imaging using Fura-2AM of iPSC-derived microglia (SFC841 line), in response to ATPγS at 50µM from baseline after pre-treatment with vehicle control or LPS (10ng/ml) + IFN-γ (10ng/ml) or 500nM PGE_2 for 24 hours. One-way ANOVA; ** $p < 0.01$; **** $p < 0.0001$.

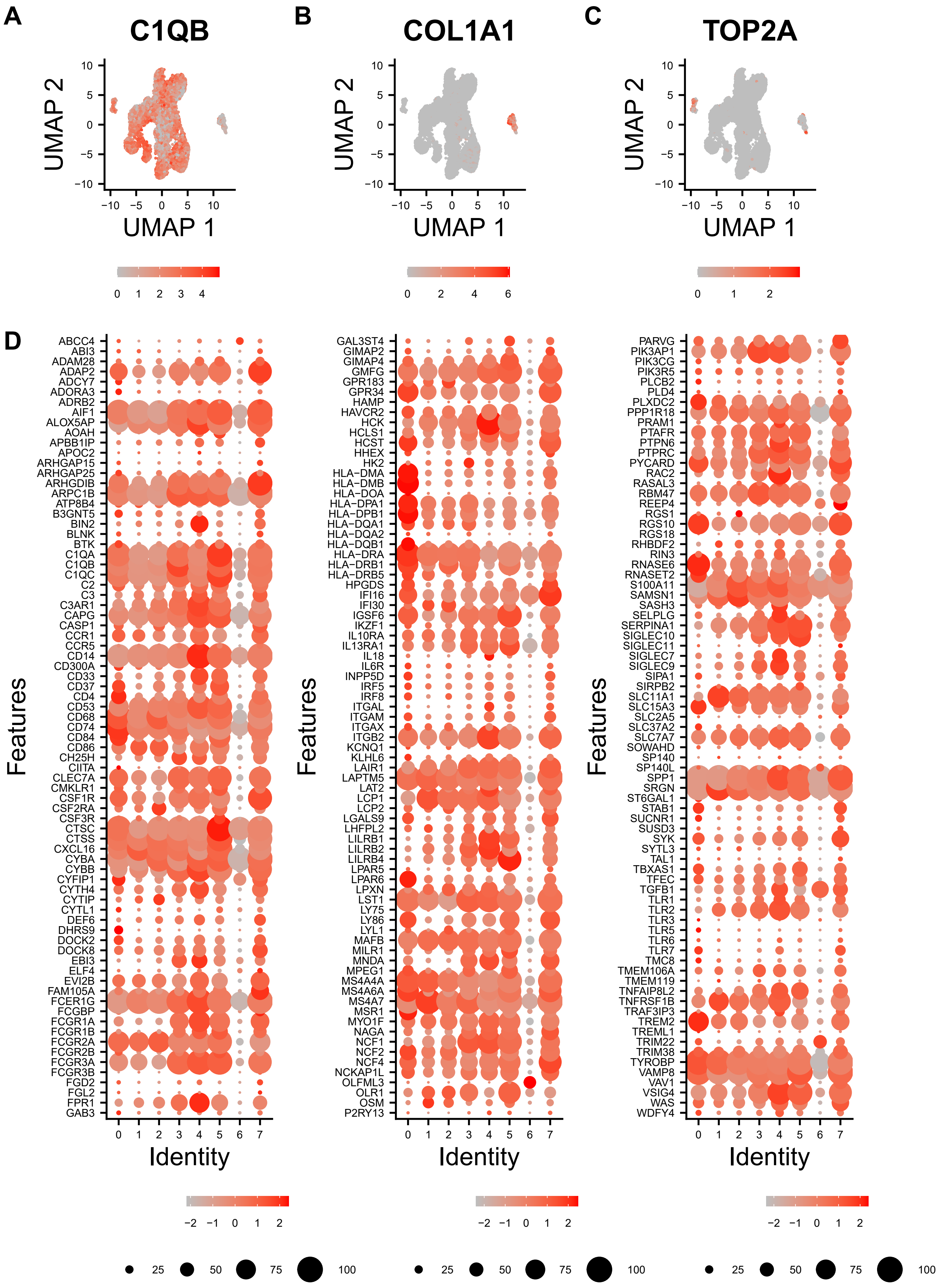

Disease Models & Mechanisms • Supplementary information

Fig. S3. Identification of microglia-like and fibroblast like cell populations. UMAP was based on the first 20 principal components of the top 1000 most variable genes across 20231 cells. **A** UMAP coloured by the expression of microglial marker C1QB. **B** UMAP coloured by the expression of COL1A1. **C.** UMAP coloured by the expression of TOP2A. **D** Dotplot show the expression patterns across cell clusters of core human microglia markers identified by Patir et al. (Patir et al.). Gene expression level is indicated by the colour while the proportion of cells where a gene is detected is represented by the size of the circles.

Disease Models & Mechanisms • Supplementary information

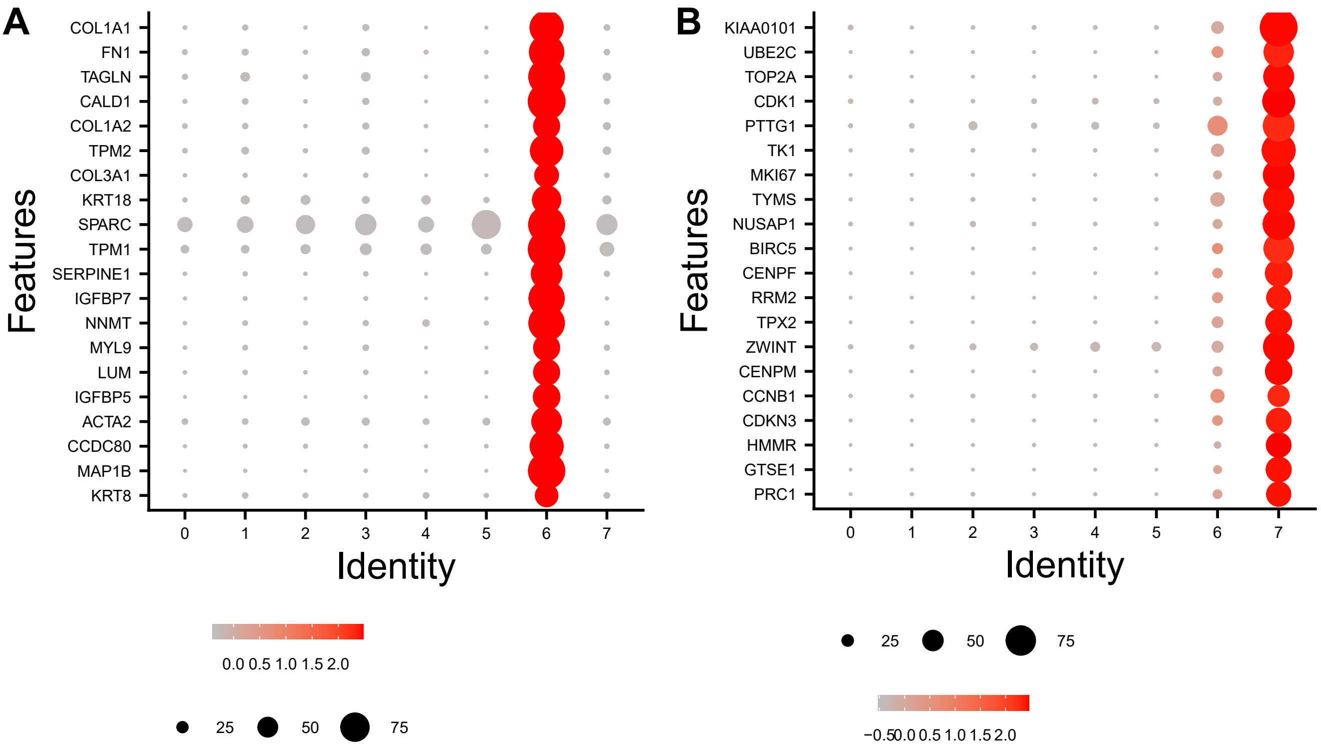

Fig. S4. Top markers of fibroblast-like and proliferating iPSC-microglia populations. A Top 20 markers of cluster 6 included genes involved in collagen fibril organization such as COL1A1 (collagen type I alpha 1 chain), COL1A2 (collagen type I alpha 2 chain), COL3A1 collagen type III alpha 1 chain and LUM (lumican) and represent a fibroblast-like population. **B** We observed genes annotated to mitotic cell cycle among the top markers of cluster 7, including TOP2A (DNA topoisomerase II alpha), TPX2 (microtubule nucleation factor), TYMS (thymidylate synthetase), UBE2C (ubiquitin conjugating enzyme E2 C), ZWINT (ZW10 interacting kinetochore protein) and RRM2 (ribonucleotide reductase regulatory subunit M2).

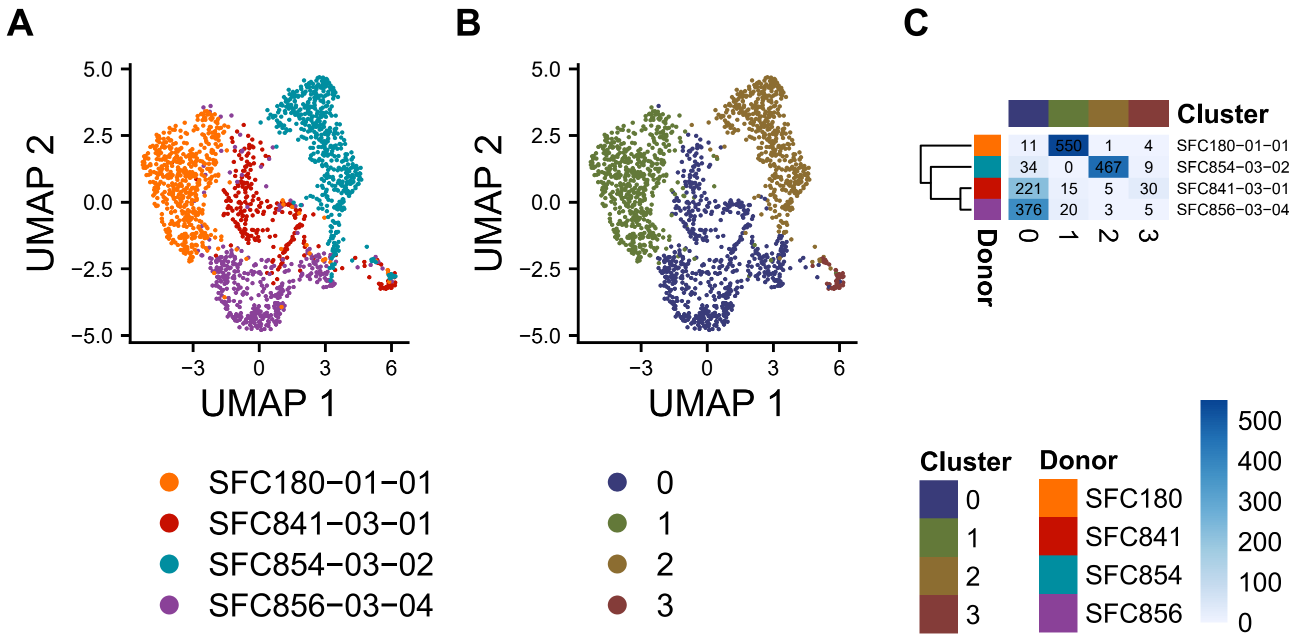

Fig. S5. Clustering in iPSC-microglia untreated control cells. We performed principal component analysis using only untreated controls, UMAP and clustering analysis were based on the first 30 principal components. **A** UMAP coloured by biological replicate/donor. **B** UMAP coloured by the clusters identified in control cells only. **C** Heatmap shows the number of cells from each donor that correspond to each of cell cluster. Cluster 0 mainly correspond to cells from donor SFC854.

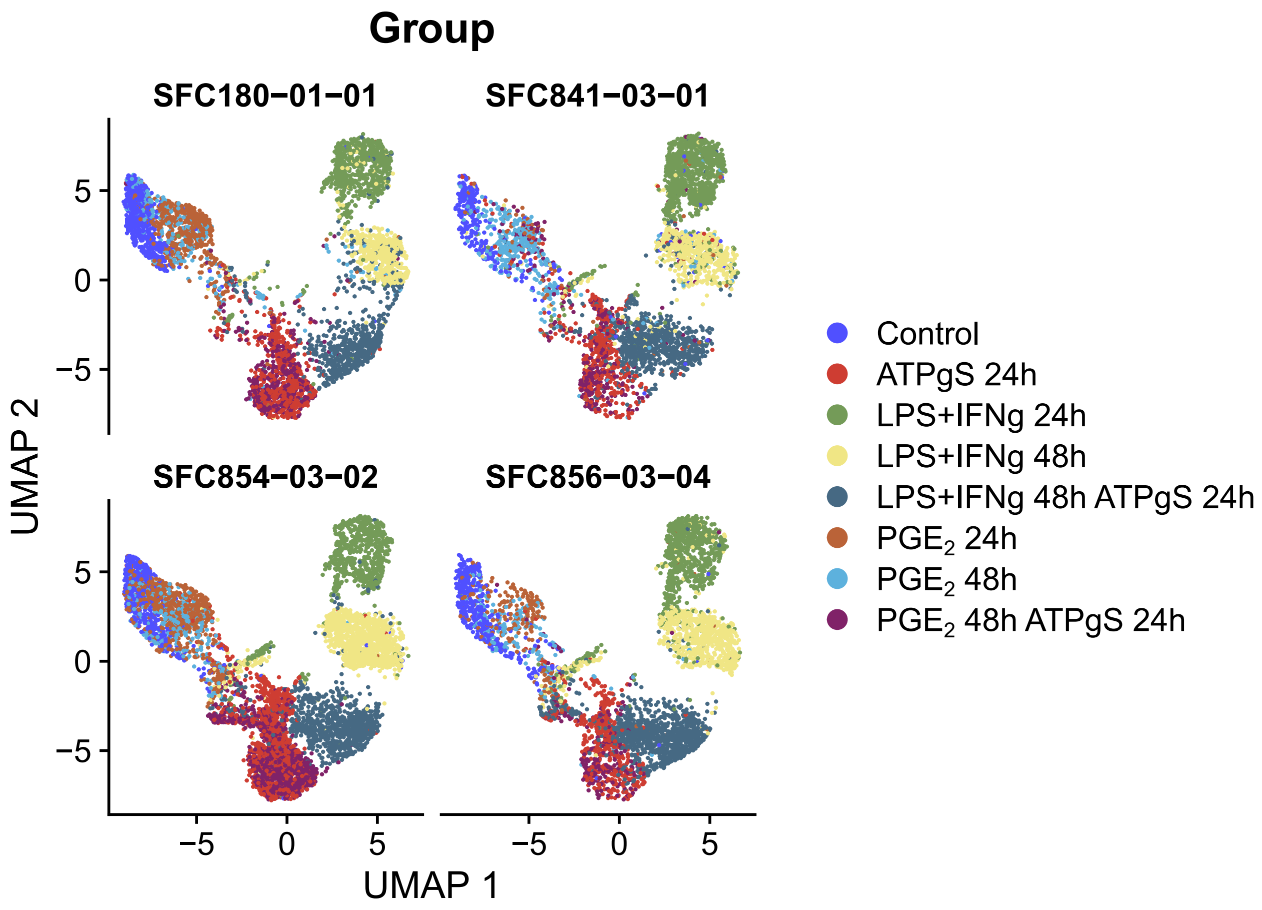

Fig. S6. Integrated iPSC-microglia across donors. Integrated gene expression data of iPSC-microglia across donors (see Methods). UMAP coloured by experimental condition and split by donor.

Disease Models & Mechanisms • Supplementary information

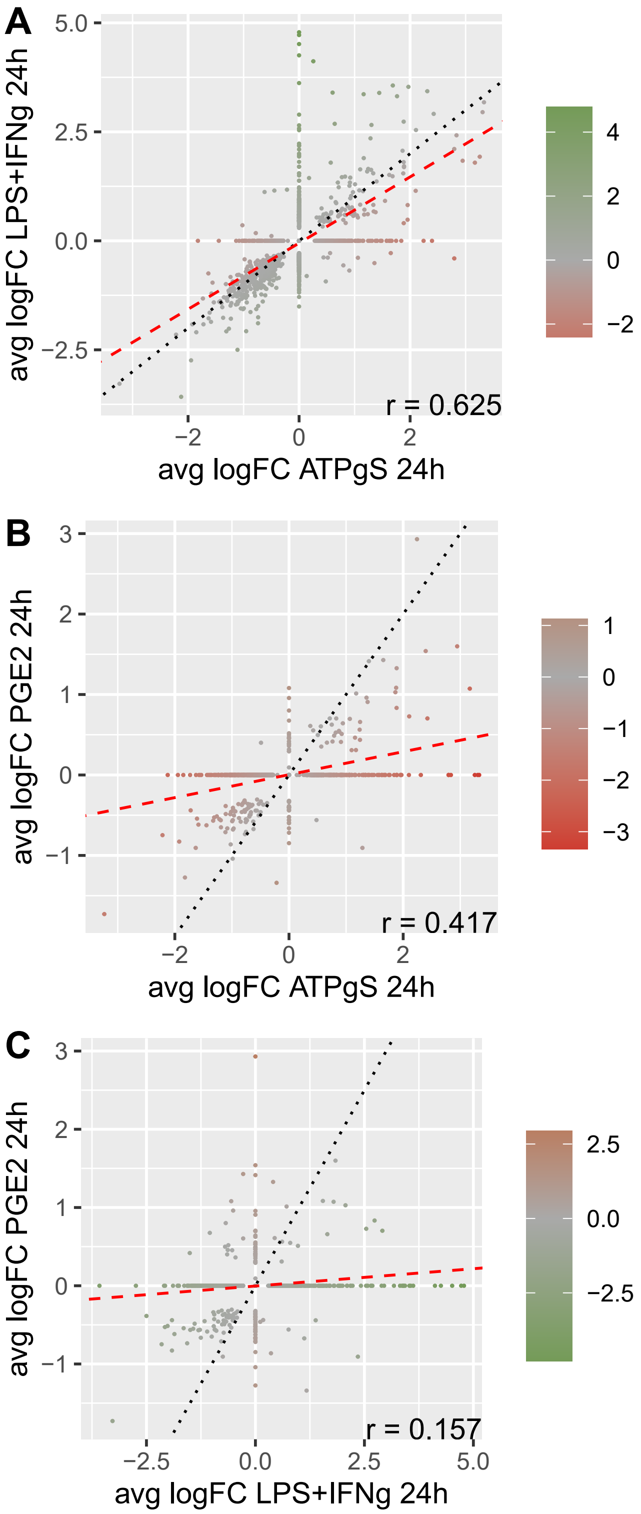

Fig. S7. Highest correlation between ATPγS and LPS+IFN-γ gene expression changes at 24h. Comparison between the log transformed fold changes (logFC) at 24h. **A** Gene expression changes in response to ATPγS and to LPS+IFN-γ at 24h. **B** Gene expression changes in response to ATPγS and to PGE2 at 24h. **C** Gene expression changes in response to LPS+IFN-γ and to PGE2 at 24h. Pearson correlation coefficient between the average logFC is indicated in the bottom right corner. Black dotted line marks equal fold changes, while red dashed line indicates the linear regression between the average log Fold Changes. Colour scheme based on the difference of the absolute fold changes and correspond to colours in Fig. 1A.

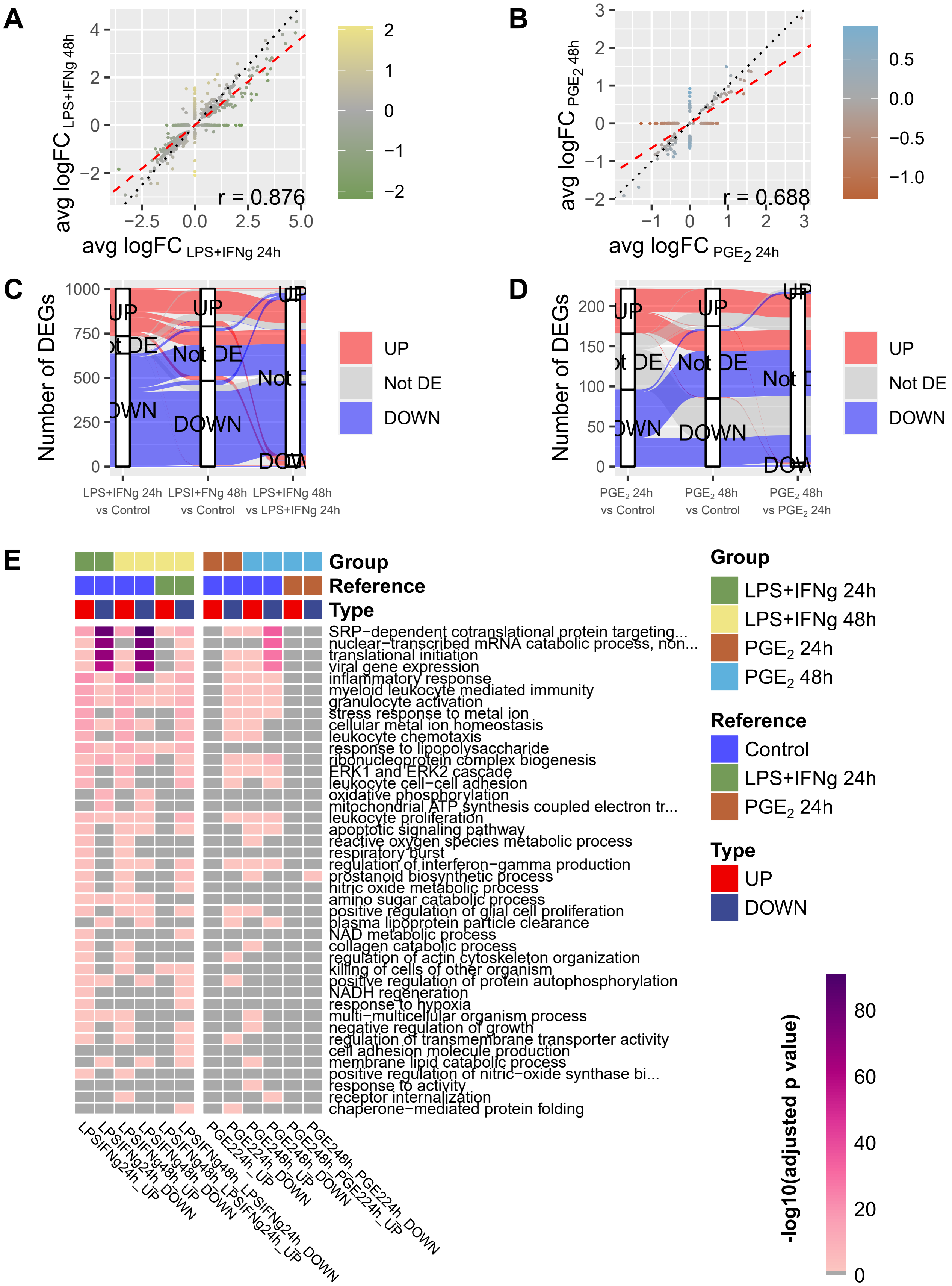

Disease Models & Mechanisms • Supplementary information

Fig. S8. Comparison of gene expression changes at 24 and 48 hours. A Comparison between the average log transformed fold changes observed at 24 and 48h after the exposure to LPS+IFN-γ. **B** Comparison between the average log transformed fold changes observed at 24 and 48h after the exposure to PGE_2. Colour scheme related to Figure 1A reflecting the condition where we observed the strongest change in expression, based on the difference of the absolute fold changes. Black dotted line marks equal fold changes, while red dashed line indicates the linear regression between the average log Fold Changes. Pearson correlation is indicated in the top left corner. **C** Sankey diagram shows DEGs in response to LPS+IFN-γ at 24 and 48h compared to control and at 48 compared to 24h. Colour indicate if genes were up or down regulated at 24h compared to control. **D.** Sankey diagram shows DEGs in response to PGE2 at 24 and 48h compared to control and at 48h compared to 24h. Similarly, colour indicate if DEGs were up or down regulated at 24h compared to control. **C** Enriched biological processes among the genes differentially expressed through time in response to either LPS+IFN-γ or PGE_2. iPSC-microglia treated with either LPS+IFN-γ or PGE_2 at 24h were compared against untreated control cells, while those exposed for 48h were compared to both the control and 24h treatment. Gene Ontology enrichment analysis of biological processes was performed separately for up and down regulated genes. Heatmap shows the transformed adjusted p value for each enriched biological process in shades of pink (if adjusted p value < 0.05, otherwise is coloured in grey).

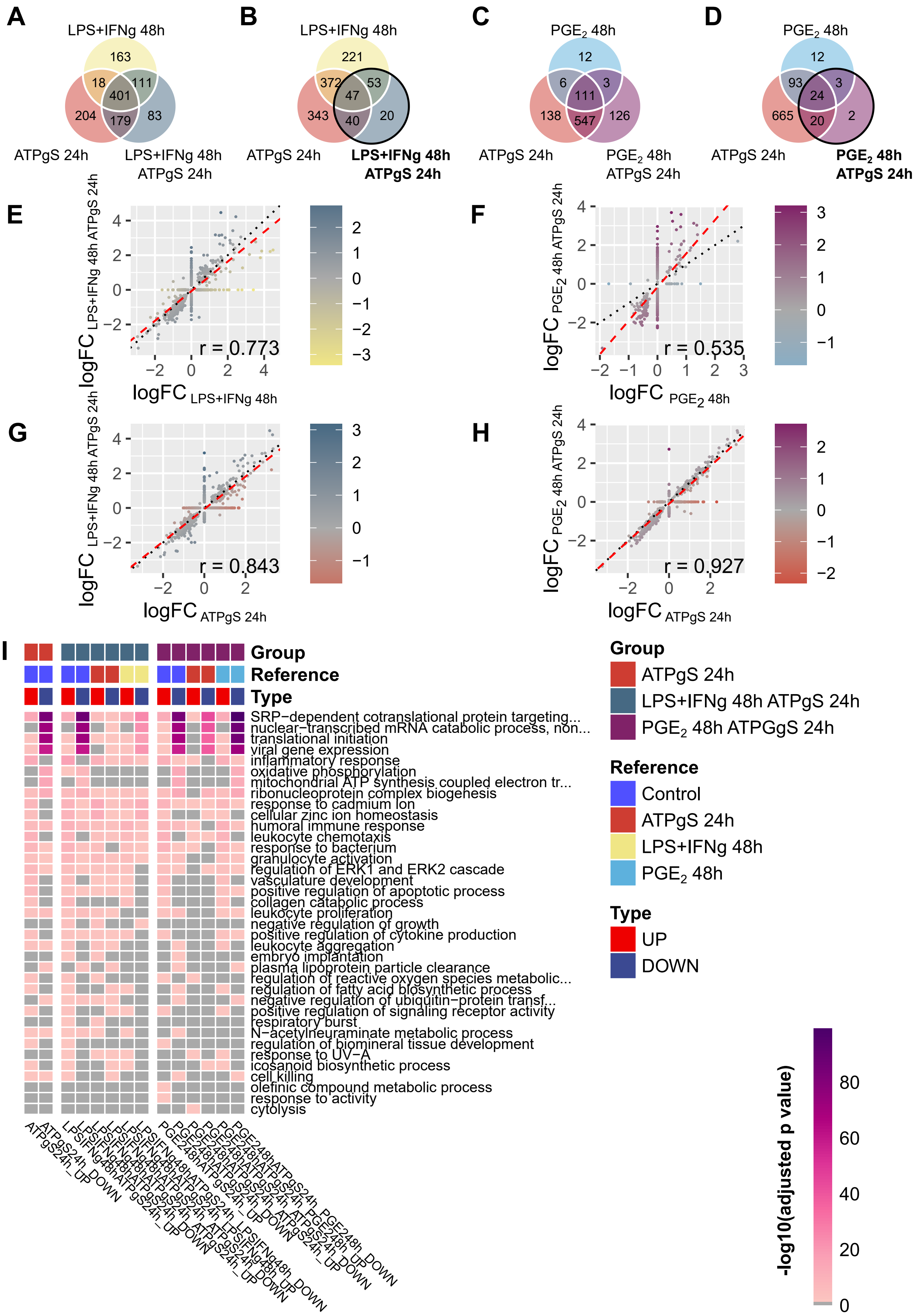

Disease Models & Mechanisms • Supplementary information

Fig. S9. Combined treatments with ATPγS. Venn diagrams show the overlap between differentially expressed genes (combined p value < 0.05): **A** Overlapping DEGs in response to LPS+IFN-γ 48h ATPγS 24h and each individual condition, in all comparisons the reference group was untreated Control cells. **B** The combined effect of LPS+IFN-γ and ATPγS was relatively small (20 genes) once we condition to the response to ATPγS. Bold and black circle indicate that the reference group for this comparison was ATPγS 24h. **C** Overlapping DEGs in response to the combined PGE_2 48h ATPγS 24h. **D** Similarly, only 2 genes were uniquely differentially expressed when exposed to both PGE_2 and ATPγS that were not detected on either of these conditions alone once we condition the response to ATPγS. Gene expression changes given as the average log Fold Change. **E** The combined treatment with LPS+IFN-γ and ATPγS compared to only LPS+IFN-γ. **F** The combined and individual treatment with PGE_2. **G** The combined treatment with LPS+IFN-γ and ATPγS compared to ATPγS only. **H** The combined treatment with PGE_2 and ATPγS compared to ATPγS alone. Colour scheme related to Figure 1A reflecting the condition where we observed the strongest change in expression, based on the difference of the absolute fold changes. Black dotted line marks equal fold changes, while red dashed line indicates the linear regression between the average log Fold Changes. Pearson correlation is indicated in the bottom right corner. **I** Enriched biological processes among the genes differentially expressed in response to ATPγS. Sets of differentially expressed genes include those in response to ATPγS alone (ATPγS 24h) and in combination with either LPS+IFN-γ treatment or PGE_2. Heatmap shows the transformed adjusted p value for each enriched biological process in shades of pink (if adjusted p value < 0.05, otherwise is coloured in grey).

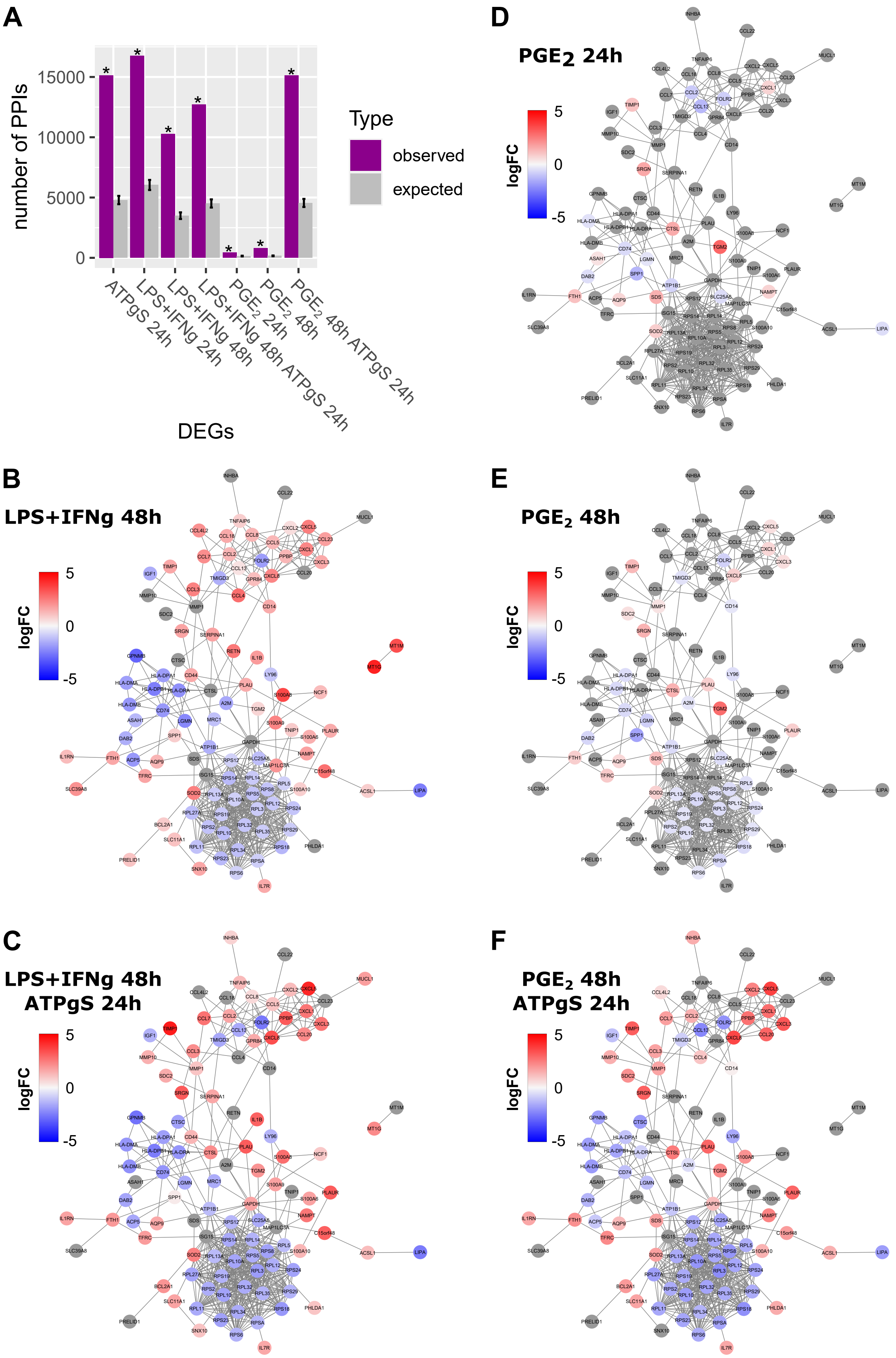

Disease Models & Mechanisms • Supplementary information

Fig. S10. Protein-protein interaction network of the genes with the strongest changes in expression in iPSC-microglia in response to the different treatments. DEGs in response to each of the different treatments in iPSC-microglia show more protein-protein interactions than expected by chance. **A** Bars shows the number of observed and expected protein-protein interaction between the gene products of DEGs in iPSC-microglia after 24 and 48 h treatment with ATPγS, LPS+IFN-γ and PGE2, as well as the combined treatments with ATPγS. Error bars show the S.E.M. based on 10,000 randomizations, * indicates an estimated p value < 0.0001. PPIs network of the corresponding DEGs in iPSC microglia with the strongest changes in expression (absolute logFC >= 1.5, combined p value < 0.05). Nodes indicate genes, edges known protein-protein interactions, and colour fold changes in response **B** LPS +IFN-γ 48h, **C** LPS+IFN-γ 48h ATPγS at 24h, **D** PGE2 24h, **E** PGE2 48h and **F** PGE2 at 48h ATPγS 24h.

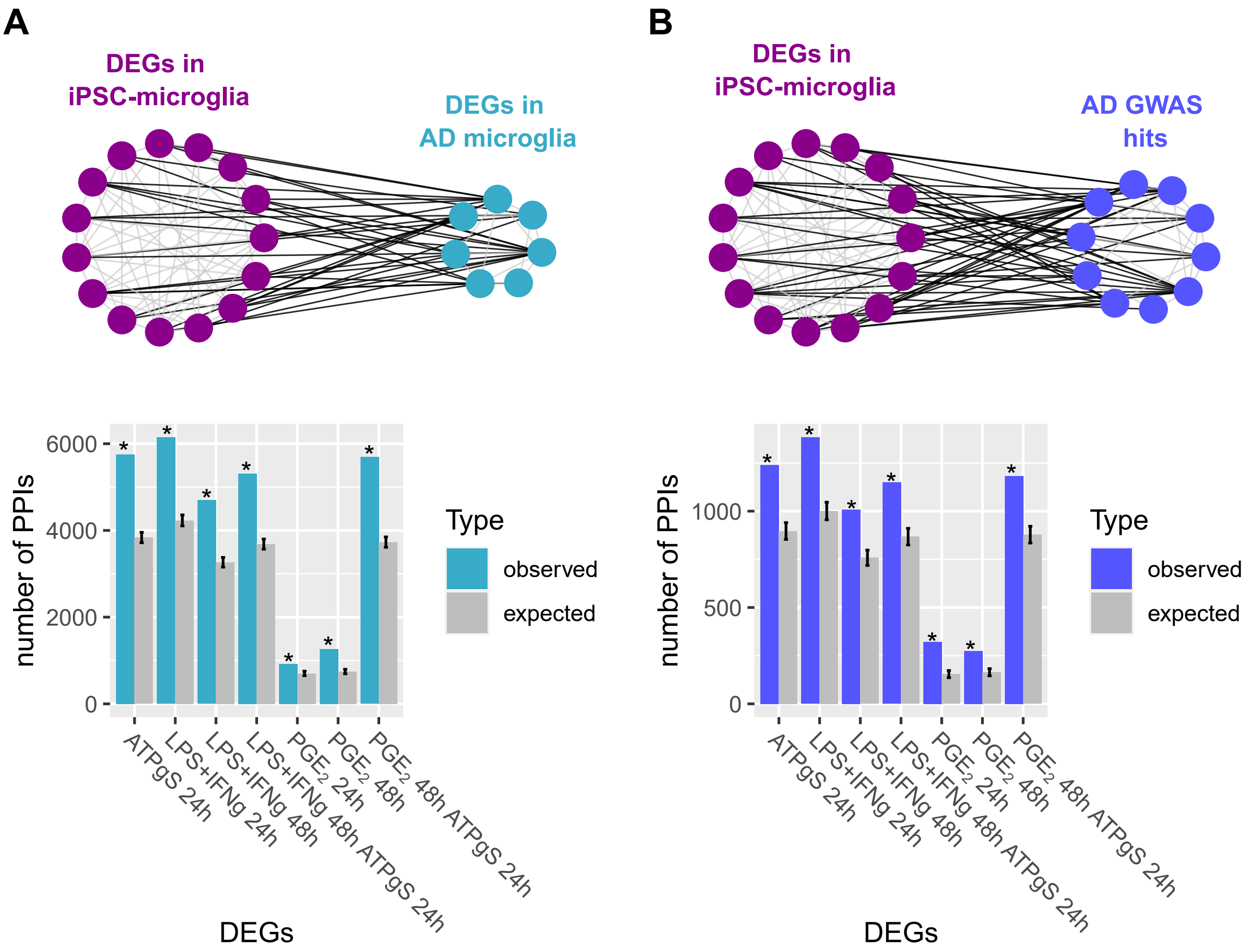

Fig. S11. Linking DEGs in iPSC-microglia to AD through protein-protein interactions. A Proteins from DEGs in stimulated iPSC-microglia show more protein-protein interactions (PPIs) to those of the DEG in microglia of AD patients. At the top the schematic representation of the protein-protein interactions between the gene products of DEGs in iSPC-microglia and those DEG in human post-mortem AD microglia. At the bottom, barplot indicates the observed and expected number of PPIs between each set of DEGs in iPSC-microglia and the protein products of the DEG in microglia from AD patients. **B** Top. Schematic representation of the protein- protein interactions (edges) between the corresponding proteins of DEGs in challenged iPSC microglia and those of genes associated to AD through GWAS. Bottom barplot indicates the number of observed and expected PPIs between DEG in iPSC-microglia and the protein products of the genes mapped to AD loci from GWAS. Error bars represent the S.E.M. based on 10,000 randomizations, * indicates an estimated p value < 0.0001.

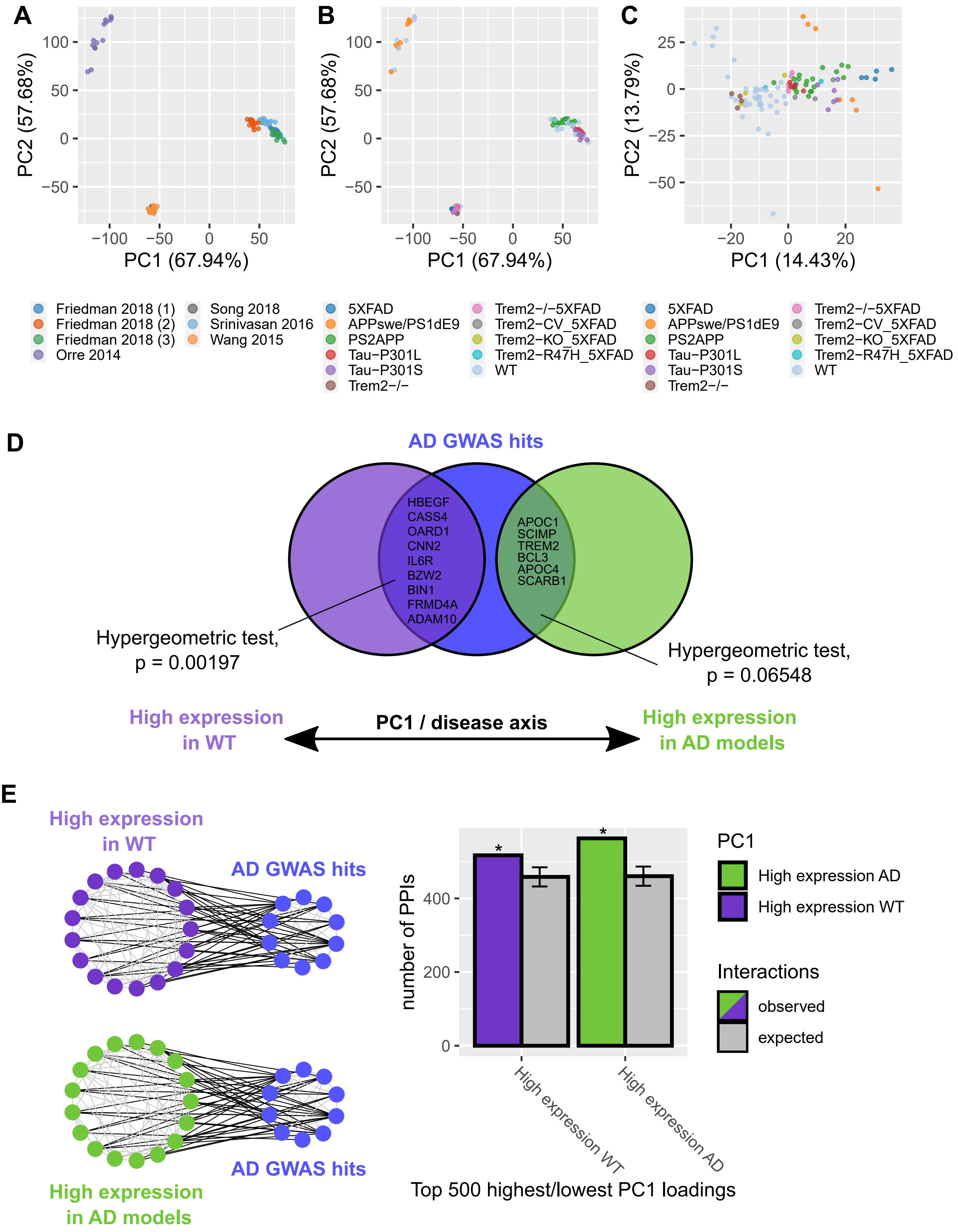
A
PC2 (57.68%)
PC1 (67.94%)
B
PC2 (57.68%)
PC1 (67.94%)
C
PC2 (13.79%)
PC1 (14.43%)
Friedman 2018 (1)
Friedman 2018 (2)
Friedman 2018 (3)
Orre 2014
Song 2018
Srinivasan 2016
Wang 2015
5XFAD
APPswe/PS1dE9
PS2APP
Tau−P301L
Tau−P301S
Trem2−/−
Trem2−/−5XFAD
Trem2−CV_5XFAD
Trem2−KO_5XFAD
Trem2−R47H_5XFAD
WT
D
AD GWAS hits
HBEGF
CASS4
OARD1
CNN2
IL6R
BZW2
BIN1
FRMD4A
ADAM10
APOC1
SCIMP
TREM2
BCL3
APOC4
SCARB1
Hypergeometric test, p = 0.00197
Hypergeometric test, p = 0.06548
High expression in WT
PC1 / disease axis
High expression in AD models
E
High expression in WT
AD GWAS hits
High expression in AD models
AD GWAS hits
number of PPIs
High expression WT
High expression AD
Top 500 highest/lowest PC1 loadings
PC1
High expression AD
High expression WT
Interactions
observed
expected

Fig. S12. Genes that drive PC1 / mouse model disease axis overlap and are connected to AD GWAS through protein-protein interactions. Principal component analysis (PCA) based on the gene expression data from multiple datasets from microglia AD mouse models before batch correction widely segregated samples by study and after by correction broadly segregated WT from transgenic mouse. **A** First two principal components before batch correction coloured by the study. **B** First two principal components before batch correction coloured by genotype. **C** First two principal components after batch correction coloured by genotype. **D** Overlap between the top 500 genes with the lowest and highest loadings along the disease axis (or highest expression in WT or AD models) and those genes associated to AD through GWAS. **E** Number of observed and expected protein-protein interactions between the top 500 driving the disease axis (either with highest or lowest loadings along PC1) and the AD GWAS hits compared to random sets of genes. Error bars represent the S.E.M. based on 10,000 randomizations,* indicates an estimated p value < 0.0001.

Disease Models & Mechanisms • Supplementary information

A

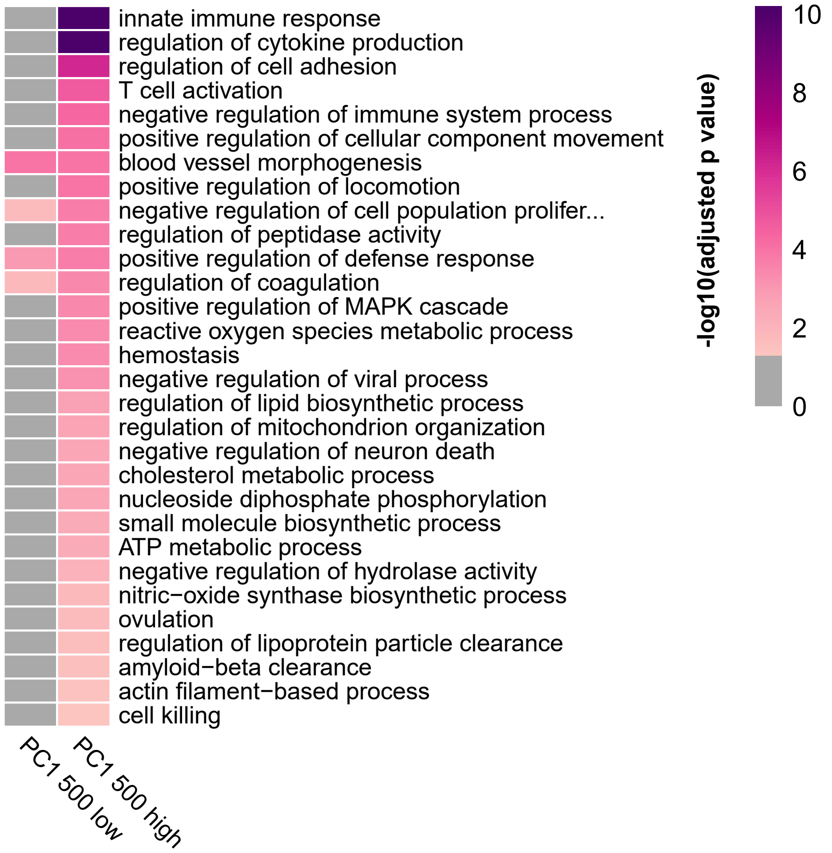
innate immune response
regulation of cytokine production
regulation of cell adhesion
T cell activation
negative regulation of immune system process
positive regulation of cellular component movement
blood vessel morphogenesis
positive regulation of locomotion
negative regulation of cell population prolifer...
regulation of peptidase activity
positive regulation of defense response
regulation of coagulation
positive regulation of MAPK cascade
reactive oxygen species metabolic process
hemostasis
negative regulation of viral process
regulation of lipid biosynthetic process
regulation of mitochondrion organization
negative regulation of neuron death
cholesterol metabolic process
nucleoside diphosphate phosphorylation
small molecule biosynthetic process
ATP metabolic process
negative regulation of hydrolase activity
nitric−oxide synthase biosynthetic process
ovulation
regulation of lipoprotein particle clearance
amyloid−beta clearance
actin filament−based process
cell killing
PC1 500 low
PC1 500 high
-log10(adjusted p value)
10
8
6
4
2
0

B

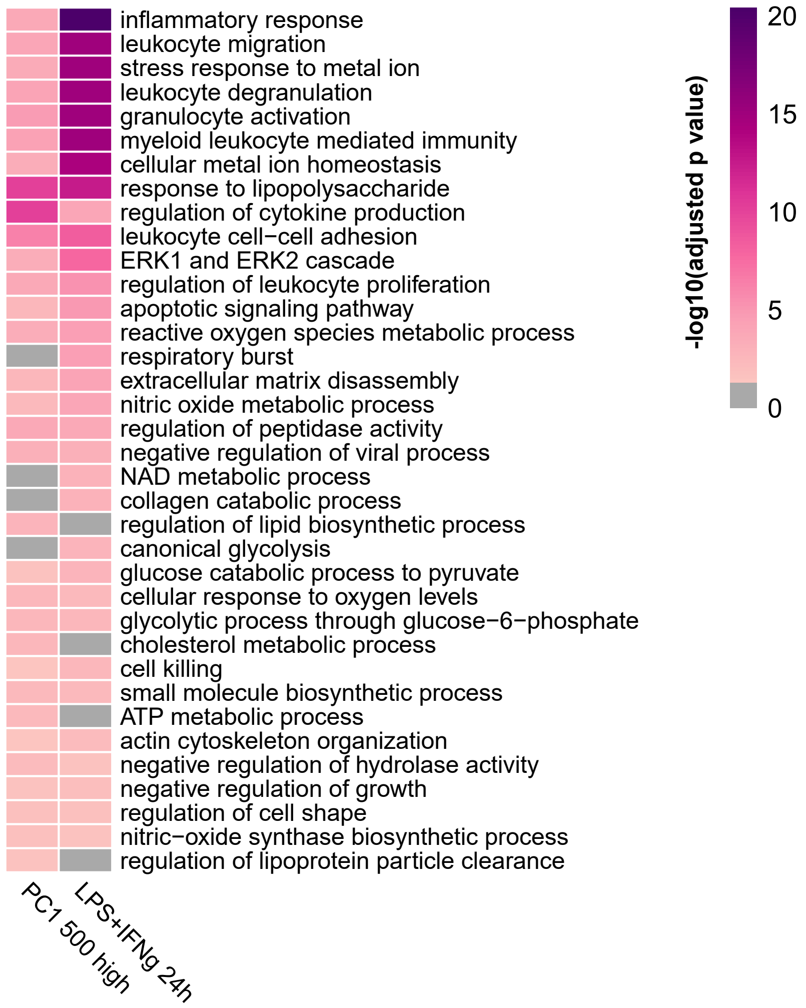
inflammatory response
leukocyte migration
stress response to metal ion
leukocyte degranulation
granulocyte activation
myeloid leukocyte mediated immunity
cellular metal ion homeostasis
response to lipopolysaccharide
regulation of cytokine production
leukocyte cell−cell adhesion
ERK1 and ERK2 cascade
regulation of leukocyte proliferation
apoptotic signaling pathway
reactive oxygen species metabolic process
respiratory burst
extracellular matrix disassembly
nitric oxide metabolic process
regulation of peptidase activity
negative regulation of viral process
NAD metabolic process
collagen catabolic process
regulation of lipid biosynthetic process
canonical glycolysis
glucose catabolic process to pyruvate
cellular response to oxygen levels
glycolytic process through glucose−6−phosphate
cholesterol metabolic process
cell killing
small molecule biosynthetic process
ATP metabolic process
actin cytoskeleton organization
negative regulation of hydrolase activity
negative regulation of growth
regulation of cell shape
nitric−oxide synthase biosynthetic process
regulation of lipoprotein particle clearance
PC1 500 high
LPS+IFNg 24h
-log10(adjusted p value)
20
15
10
5
0

Fig. S13. Functional characterization of genes along the disease axis and convergence in functional pathways with the genes up regulated in response to LPS+IFN-γ in iPSC-microglia at 24h. A Gene Ontology enrichment analysis among the top 500 genes with the highest and lowest loadings along the disease axis (PC1 from mouse meta-analysis). GO terms were reduced to a non-redundant set based on the semantic similarity in mouse. **B** We observed a strong pathway convergence from those genes driving the disease axis (top 500 highest PC1 loadings) and among the DEGs upregulated in response to LPS+IFN-γ at 24h. Heatmap shows the minus log transformed p value for the enriched biological processes among each set of genes (shades of pink if adjusted p value < 0.05, grey otherwise).

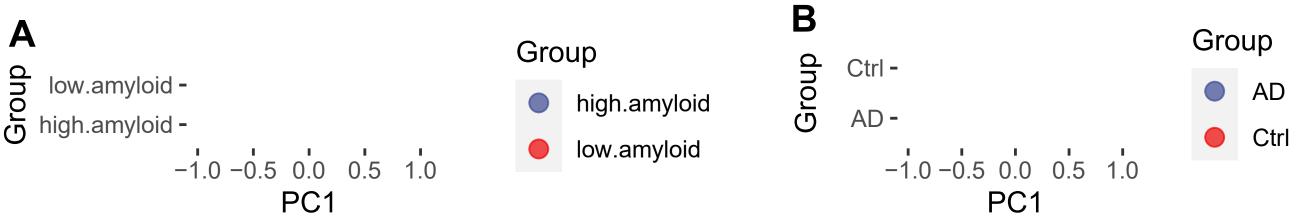

Fig. S14. Post-mortem microglia from AD show a minor shift along disease axis based on genetic mouse models of AD. A Single cell gene expression from human post-mortem microglia from the prefrontal cortex was aggregated from 24 controls (low β amyloid pathology) and AD cases (high β amyloid) reduce to one to one human to mouse orthologs and projected into the first principal component from the mouse meta-analysis. **B** Single cell gene expression from human post-mortem microglia from the entorhinal cortex was aggregated from 6 AD cases and 6 controls, reduced to one to one ortholog genes from human to mice and projected into the disease axis from the meta-analysis of mouse disease models of AD.

Disease Models & Mechanisms • Supplementary information

Table S1. QC metrics from Picard for mouse microglia RNA-seq datasets included in the meta-analysis. All libraries were single-end, un stranded and 50bp in length. Seq-bias and GC-bias correction used in Salmon version 0.9.1.

Study ID	Sample ID	reads processed (transcriptome)	reads mapped (transcriptome)	percent mapped (transcriptome)	PCT CODING BASES	PCT UTR BASES	PCT INTRONIC BASES	PCT INTERGENIC BASES	PCT MRNA BASES	PCT USABLE BASES	MEDIAN CV COVERAGE	MEDIAN 5PRIME BIAS	MEDIAN 3PRIME BIAS	MEDIAN 5PRIME TO 3PRIME BIAS	PCT GC
GSE75431	GSM1955013	25419397	6161867	24.24	12.6	34.9	40.25	12.3	47.5	42.6	0.82	0.2	0.94	0.2	45
GSE75431	GSM1955017	32452939	7614699	23.46	13.3	43.1	33.77	9.91	56.3	51.1	0.81	0.21	0.82	0.24	44
GSE75431	GSM1955023	39738697	7176767	18.06	12.1	57.2	17.22	13.52	69.3	61.3	0.89	0.22	0.4	0.32	46
GSE75431	GSM1955025	26749355	4211472	15.74	10.1	65.9	15.19	8.81	76	68.5	0.88	0.24	0.45	0.27	42
GSE75431	GSM1955028	44485248	9902601	22.26	12.3	43.5	32.35	11.78	55.9	49.4	0.84	0.19	0.82	0.22	44
GSE75431	GSM1955031	17495157	2205558	12.61	7.89	76.4	7.51	8.17	84.3	77	1.02	0.15	0.53	0.1	40
GSE75431	GSM1955035	16096484	3286593	20.42	13.4	56.8	21.08	8.76	70.2	62.1	0.93	0.18	0.49	0.2	43
GSE75431	GSM1955036	30765692	3160247	10.27	5.37	73.9	8.18	12.57	79.3	69.2	1	0.16	0.59	0.12	42
GSE75431	GSM1955037	25842508	4036037	15.62	8.69	55.1	20.34	15.83	63.8	55.4	0.88	0.23	0.63	0.23	47
GSE75431	GSM1955039	24778357	4694399	18.95	11	54.2	28.6	6.28	65.1	60	0.86	0.21	0.56	0.3	40
GSE75431	GSM1955041	28044077	6194662	22.09	13	44.5	30.39	12.13	57.5	51.2	0.78	0.24	0.73	0.32	46
GSE75431	GSM1955045	32641862	4861202	14.89	8.96	65.8	16.32	8.88	74.8	66.9	0.95	0.2	0.53	0.23	42
GSE75431	GSM1955048	33085464	4045050	12.23	7.23	68.5	10.39	13.91	75.7	66.5	0.94	0.19	0.52	0.16	45
GSE75431	GSM1955053	27379120	6803057	24.85	14.9	41.4	34.43	9.23	56.3	50.5	0.85	0.19	0.68	0.22	44
GSE75431	GSM1955056	39015585	10422531	26.71	15.8	39.7	33.95	10.53	55.5	50.5	0.8	0.2	0.68	0.31	44
GSE75431	GSM1955059	30326170	5464730	18.02	11.2	61.2	18.89	8.7	72.4	65.7	0.9	0.21	0.53	0.29	42
GSE75431	GSM1955060	28950409	6404664	22.12	12.4	47.9	28.37	11.28	60.4	54.4	0.87	0.18	0.86	0.19	44
GSE75431	GSM1955064	18789113	4720657	25.12	14.9	45	26.57	13.56	59.9	53.7	0.86	0.17	0.87	0.17	43
GSE75431	GSM1955065	41206159	9123281	22.14	12.2	41.6	38.05	8.17	53.8	49	0.84	0.21	0.67	0.29	43
GSE75431	GSM1955067	43873000	11972003	27.29	15.5	34	39.48	10.96	49.6	44.6	0.83	0.2	0.83	0.23	45
GSE89482	GSM2373561	24806418	6661389	26.85	9.67	24	59.01	7.31	33.7	29.3	0.93	0.09	1.48	0.06	41
GSE89482	GSM2373562	27969338	7771358	27.79	10.3	22.5	60.05	7.24	32.7	29	0.91	0.08	1.38	0.06	42
GSE89482	GSM2373563	29680769	8602219	28.98	8.52	25	58.88	7.57	33.6	30.2	0.91	0.06	1.51	0.05	38
GSE89482	GSM2373564	27348945	8133030	29.74	11.1	22.3	60.08	6.48	33.4	31.2	0.8	0.1	1.09	0.09	41
GSE89482	GSM2373565	22653489	5006860	22.1	7.5	19.2	66.57	6.7	26.7	24.2	0.8	0.12	1.07	0.12	40
GSE89482	GSM2373566	24688240	7563532	30.64	10.9	25.3	57.37	6.44	36.2	33.5	0.76	0.1	1.12	0.09	40
GSE89482	GSM2373567	23391977	6494318	27.76	7.66	23.9	61.31	7.13	31.6	27.6	1.02	0.05	1.63	0.03	38
GSE89482	GSM2373568	28610058	7449680	26.04	9.4	24.3	60.15	6.19	33.7	30.8	0.79	0.09	1.13	0.08	41
GSE89482	GSM2373569	29761798	9844106	33.08	13	28.8	51.52	6.77	41.7	38.9	0.78	0.1	1.06	0.09	41
GSE89482	GSM2373570	27687168	8004452	28.91	9.73	23.4	59.79	7.12	33.1	29.6	0.82	0.09	1.28	0.07	40
GSE89482	GSM2373571	28927936	7307237	25.26	7.89	20.6	64.73	6.81	28.5	25.8	0.96	0.05	1.29	0.03	40
GSE89482	GSM2373572	28906773	7700732	26.64	9.01	20.9	63.23	6.86	29.9	27.2	0.83	0.1	1.34	0.08	41
GSE89482	GSM2373573	37322100	11075490	29.68	9.9	25.2	57.53	7.36	35.1	32.5	0.75	0.1	1.11	0.1	39
GSE89482	GSM2373574	21871815	5359892	24.51	7.58	20.1	65.25	7.03	27.7	24.3	0.99	0.07	1.68	0.05	40
GSE89482	GSM2373575	29146258	6709197	23.02	7.13	18	67.64	7.25	25.1	22.3	0.91	0.11	1.3	0.08	39
GSE93179	GSM2445938	36739457	6546718	17.82	9.86	44.1	36.51	9.56	53.9	42	1.45	0.07	0.66	0.05	39
GSE93179	GSM2445939	23671070	4625515	19.54	11.2	30	48.38	10.38	41.2	31.1	1.33	0.09	0.55	0.09	41
GSE93179	GSM2445940	28093434	5222219	18.59	10.1	36	43.13	10.73	46.1	33.5	1.4	0.07	0.61	0.05	42
GSE93179	GSM2445941	24961057	4625414	18.53	11.1	27.4	51.79	9.74	38.5	26.6	1.34	0.08	0.8	0.07	41
GSE93179	GSM2445942	27419413	4540753	16.56	7.64	29	54.77	8.64	36.6	26.9	1.42	0.06	1.03	0.03	38
GSE93179	GSM2445943	21239262	3863710	18.19	9.2	26.8	53.35	10.7	36	28.4	1.31	0.09	0.59	0.08	39
GSE93179	GSM2445944	26853626	5958491	22.19	11.4	30.5	43.04	15	42	32.9	1.77	0.01	0.55	0	35
GSE93179	GSM2445945	19720950	3775889	19.15	10.8	38.7	41.63	8.88	49.5	40.7	1.33	0.09	0.45	0.09	38
GSE93179	GSM2445946	16654901	3484053	20.92	9.02	22.8	55.31	12.88	31.8	25.4	1.55	0.03	0.5	0	38
GSE93180	GSM2445947	21729913	4208499	19.37	9.2	47.4	35.6	7.8	56.6	49.8	1.16	0.05	0.95	0.01	38
GSE93180	GSM2445948	27747633	7184886	25.89	12	31.3	41.86	14.89	43.3	35.6	1.25	0.05	0.46	0.01	39
GSE93180	GSM2445949	36632134	7300810	19.93	11.2	52.3	29.89	6.63	63.5	56.7	1.08	0.07	0.46	0.03	39
GSE93180	GSM2445950	31803938	10432206	32.8	16.6	27.5	32.14	23.77	44.1	33.9	1.15	0.06	0.54	0.03	38
GSE93180	GSM2445951	37390596	14945449	39.97	20.8	12.7	31.32	35.16	33.5	24.2	1.41	0	0.68	0	37
GSE93180	GSM2445952	29834525	10870683	36.44	17.5	13.4	36.38	32.65	31	22.8	1.37	0.01	0.8	0	36
GSE93180	GSM2445953	26218447	6747999	25.74	14.3	44.9	26.31	14.55	59.2	48.3	1.18	0.07	0.59	0.02	37
GSE93180	GSM2445954	26831207	6023345	22.45	11.5	40.5	27.44	20.5	52.1	43.2	1.35	0	0.45	0	37
GSE93180	GSM2445955	26781529	8600320	32.11	14.9	17.2	36.09	31.78	32.1	24.4	1.32	0.02	0.44	0	38
GSE93180	GSM2445956	29428746	5813514	19.75	11	56.1	24.37	8.54	67.1	58.9	1.13	0.06	0.7	0.02	39
GSE93180	GSM2445957	32661092	10640484	32.58	16.1	23.4	43.54	16.99	39.5	32	1.09	0.08	0.48	0.06	39
GSE93180	GSM2445958	31603131	5815390	18.4	9.52	53.3	29.28	7.92	62.8	55.7	1.08	0.09	0.58	0.05	39

Disease Models & Mechanisms • Supplementary information
